# Developmental trajectories of thalamic progenitors revealed by single-cell transcriptome profiling and Shh perturbation

**DOI:** 10.1016/j.celrep.2022.111768

**Published:** 2022-12-06

**Authors:** Kiya W. Govek, Sixing Chen, Paraskevi Sgourdou, Yao Yao, Steven Woodhouse, Tingfang Chen, Marc V. Fuccillo, Douglas J. Epstein, Pablo G. Camara

**Affiliations:** 1 Department of Genetics, Perelman School of Medicine, University of Pennsylvania, 3700 Hamilton Walk, Philadelphia, PA 19104, USA; 2 Institute for Biomedical Informatics, Perelman School of Medicine, University of Pennsylvania, 3700 Hamilton Walk, Philadelphia, PA 19104, USA; 3 Department of Animal and Dairy Science, Regenerative Bioscience Center, University of Georgia, 425 River Road, Athens, GA 30602, USA; 4 Department of Neuroscience, Perelman School of Medicine, University of Pennsylvania, Philadelphia, PA 19104, USA; 5 These authors contributed equally; 6 Lead contact

## Abstract

The thalamus is the principal information hub of the vertebrate brain, with essential roles in sensory and motor information processing, attention, and memory. The complex array of thalamic nuclei develops from a restricted pool of neural progenitors. We apply longitudinal single-cell RNA sequencing and regional abrogation of Sonic hedgehog (Shh) to map the developmental trajectories of thalamic progenitors, intermediate progenitors, and post-mitotic neurons as they coalesce into distinct thalamic nuclei. These data reveal that the complex architecture of the thalamus is established early during embryonic brain development through the coordinated action of four cell differentiation lineages derived from Shh-dependent and -independent progenitors. We systematically characterize the gene expression programs that define these thalamic lineages across time and demonstrate how their disruption upon Shh depletion causes pronounced locomotor impairment resembling infantile Parkinson’s disease. These results reveal key principles of thalamic development and provide mechanistic insights into neurodevelopmental disorders resulting from thalamic dysfunction.

## In brief

Govek et al. describe the developmental trajectory of thalamic nuclei from Shh-dependent and -independent neural progenitors. They show that molecular signatures of thalamic neuronal subtypes can be distinguished prior to their aggregation into histologically distinct thalamic nuclei and that alterations in Shh cause neurodevelopmental disorders attributed to thalamic dysfunction.

## Graphical Abstract

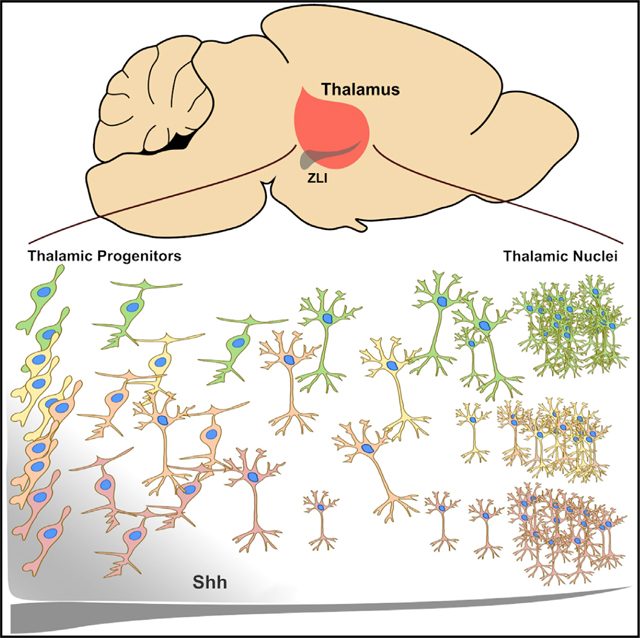

## INTRODUCTION

The thalamus develops in the posterior region of the diencephalon, between the mesencephalon and telencephalon. This location is important for unique aspects of thalamic function, to process and relay sensory and motor information to and from the cerebral cortex, and to regulate sleep, alertness, and consciousness.^1^ How the thalamus comes to reside within this region of the central nervous system (CNS) has been the subject of much investigation.^2,3^ Extracellular signals secreted from key locations both extrinsic and intrinsic to the thalamic primordium have been shown to play important roles in the growth, regionalization, and specification of thalamic progenitors.^4–16^ One factor in particular, the secreted morphogen Sonic hedgehog (Shh), has been implicated in spatiotemporal and threshold models of thalamic development that differ from other areas of the CNS. This impact of Shh is due, in large part, to its expression within two signaling centers, the basal plate and the zona limitans intrathalamica (ZLI), a dorsally projecting spike that separates the thalamus from the prethalamic territory.^17,18^ Shh signaling from these sources exhibits both unique and overlapping functions in the control of thalamic progenitor identity and specification of thalamic nuclei.^11–13^

The adult thalamus is subdivided into 44 distinct nuclei that are categorized on the basis of their positioning (anterior, medial, lateral, ventral, posterior, and intralaminar groups), cytoarchitectural properties, types of input (sensory, motor, or limbic), and hierarchical connectivity patterns to and from the cerebral cortex and subcortical areas.^1,19–25^ The spatial arrangement of these nuclei is important for generating the precise topographical relationship needed to fulfill its role as a relay and information processing center. Despite advances in our understanding of the early events regulating thalamic growth and regionalization, there remain major gaps in knowledge of the mechanisms by which heterogeneous clusters of mostly excitatory relay neurons are specified and aggregate into distinct thalamic nuclei. One particular challenge has been to decipher the full complement of thalamic progenitor identities and to elucidate their contribution to specific thalamic nuclei.^11,12,26–30^ Thus far, only two distinct thalamic progenitor domains have been defined by gene expression and fate mapping studies. The caudal population of thalamic progenitors, cTh.Pro, gives rise to all glutamatergic thalamic nuclei that extend axonal projections to the neocortex.^28^ The rostral population of thalamic progenitors, rTh.Pro, comprises a narrow band of cells sandwiched between cTh.Pro and the ZLI. Thalamic neurons derived from rTh.Pro are GABAergic and contribute to the ventrolateral geniculate nucleus (VLG) and the intergeniculate leaflet (IGL).^1,12,28,31–33^

Multiple studies have shown that thalamic nuclei exhibit extensive heterogeneity at the level of gene expression.^2,25,34–38^ Nevertheless, we still lack a clear understanding of the molecular logic and developmental trajectories by which thalamic nuclei acquire their distinct identities. Here, we make use of highly parallelized single-cell RNA sequencing (RNA-seq) to molecularly and anatomically characterize thalamic progenitor subtypes, intermediate progenitors, and post-mitotic neurons across multiple stages of mouse embryonic development. Our approach overcomes limitations of conventional single-cell transcriptomic atlases, which often lack mechanistic detail, by investigating how regional abrogation of Shh expression alters thalamic lineage progression at single-cell scale. Our findings unify models of thalamic development and provide a detailed understanding of a neurodevelopmental disorder resulting from alterations in thalamic architecture.

## RESULTS

### Deletion of SBE1 and SBE5 abrogates *Shh* expression in the ZLI

**Figure 1.**
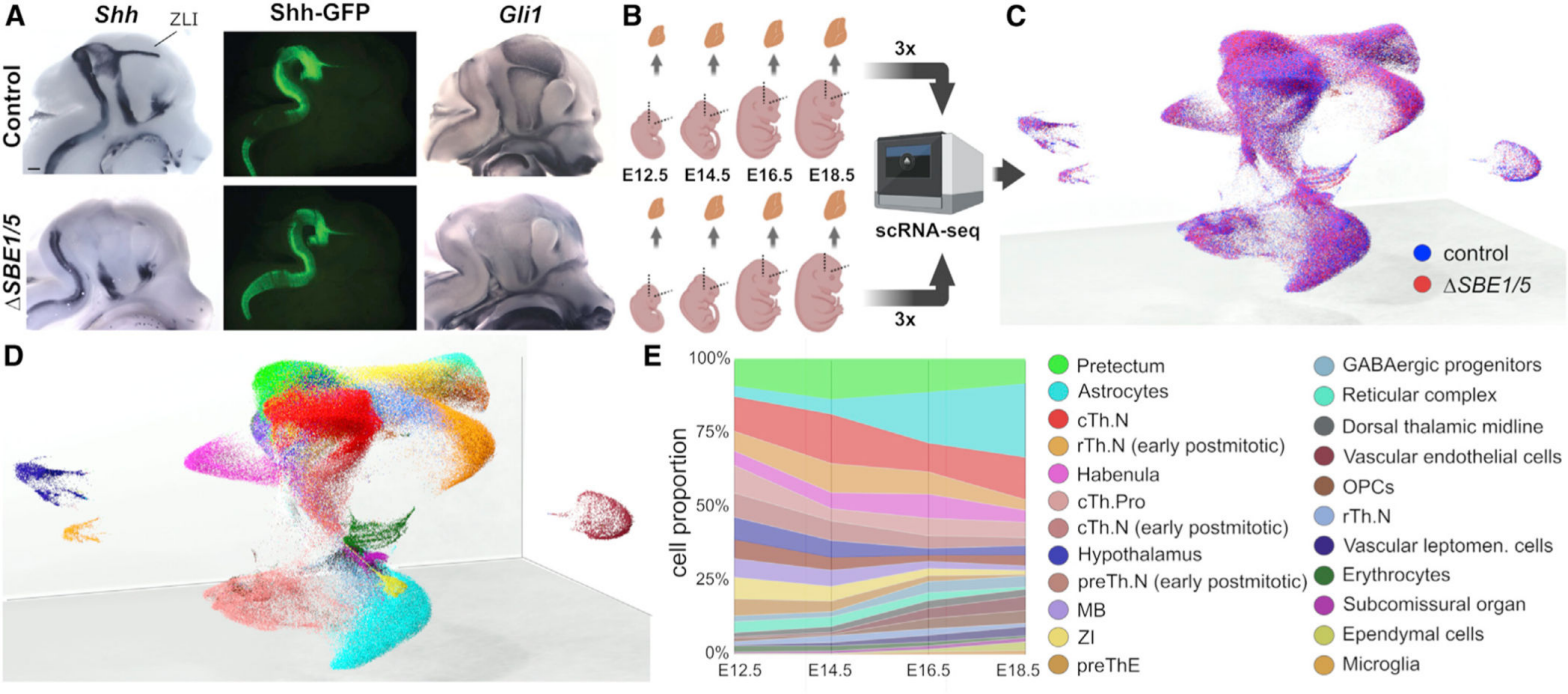
A high-resolution single-cell transcriptomic atlas of the developing caudal diencephalon (A) Deletion of the SBE1 and SBE5 enhancers leads to a loss of *Shh* expression and Shh signaling activity in the caudal diencephalon. Whole-mount RNA *in situ* hybridization for *Shh* (left) and the Shh-responsive gene *Gli1* (right) in bisected heads from control (top) and *ΔSBE1/5* embryos (bottom) at E12.5 shows reduced expression in the caudal diencephalon of mutant embryos (n = 3). Scale bar, 500 μm. A Shh bacterial artificial chromosome (BAC) transgene (Shh-GFP) expressing eGFP in place of Shh shows persistent reporter activity in the ZLI of *ΔSBE1/5* and control embryos (center). (B) Schematic of the experimental study design. The caudal diencephalon of control and *ΔSBE1/5* embryos was micro-dissected at E12.5, E14.5, E16.5, and E18.5 in three replicates per time point and genotype, fixed in methanol, and profiled with single-cell RNA-seq. (C) 3D UMAP representation of the consolidated single-cell gene expression space across all samples, colored by the genotype of the cells. Cells from control and *ΔSBE1/5* embryos substantially overlap in the representation. (D) 3D UMAP representation of the single-cell transcriptomic atlas colored by the 23 cell populations identified in the clustering analyses. (E) Fraction of cells in each cell population for each of the four time points. Only cells from control mice were considered in this analysis. The observed expansion of glial cells starting between E14.5 and E16.5 is consistent with the transition between neurogenesis and gliogenesis at this stage of embryonic development. Cell populations are listed in the legend in the same order as they appear in the band plot. cTh.N, caudal thalamic neurons; rTh.N, rostral thalamic neurons; cTh.Pro, caudal thalamic progenitors; preTh.N, prethalamic neurons; MB, midbrain; ZI, zona incerta; preThE, prethalamic eminence; OPCs, oligodendrocyte precursor cells. See also Figure S1 and Tables S1 and S2.

*Shh* expression in the ZLI and basal plate of the caudal diencephalon is dependent on two Shh brain enhancers: SBE1 and SBE5.^39^ Mouse embryos homozygous for targeted deletions of SBE1 and SBE5 (*Shh*^*ΔSBE1ΔSBE5/ΔSBE1ΔSBE5*^, herein referred to as *ΔSBE1/5*) fail to activate *Shh* transcription in the ZLI and basal plate after E10.0, compared with control littermates (*Shh*^*ΔSBE1ΔSBE5/+*^) (Figure 1A).^39^ Consequently, Shh signaling activity in thalamic and prethalamic territories is compromised in *ΔSBE1/5* embryos, as indicated by the loss of *Gli1* expression (Figure 1A). Despite the absence of *Shh* expression and Shh signaling activity, a GFP reporter transgene driven by SBE1 continued to be expressed in the ZLI of *ΔSBE1/5* embryos (Figure 1A). Moreover, genes coding for transcription factors that are normally expressed in the ZLI, such as *Pitx2* and *Foxa1*, maintained much of their expression in *ΔSBE1/5* mutants, albeit in a partially reduced area (Figure S1A). These results indicate that the cellular integrity of the ZLI remains intact in *ΔSBE1/5* embryos and highlight the utility of this mouse model for studying the implications of Shh signaling in mammalian thalamic development.

### A high-resolution single-cell transcriptomic atlas of the developing caudal diencephalon in control and *ΔSBE1/5* embryos

To uncover the molecular logic driving the specification of distinct thalamic nuclei, we generated a high-resolution single-cell RNA-seq atlas of the developing caudal diencephalon in control and *ΔSBE1/5* embryos. The thalamic primordia were micro-dissected from three embryos per genotype according to anatomical landmarks (STAR Methods) at four developmental stages (E12.5, E14.5, E16.5, and E18.5) coinciding with peak periods of thalamic proliferation, neurogenesis, and differentiation (Figure 1B; n = 24 embryos in total). Overall, we captured the transcriptome of 249,071 cells (121,878 cells from control and 127,193 cells from *ΔSBE1/5* embryos) with a median of 2,466 genes and 4,891 unique molecular identifiers (UMIs) detected per cell. We consolidated the single-cell gene expression space of all the samples and clustered the cells in this space (Figures 1C and 1D).

Our analysis identified 23 distinct cell populations, comprising most of the known cell types in the caudal diencephalon (Figures 1D and 1E). Cells from three independent biological replicates overlapped in the consolidated representation and were similarly delineated across all cell populations (Figure S1B), indicating the absence of large batch effects. Most of the identified cell populations were part of continuous developmental trajectories, such as the differentiation of thalamic progenitors (Th.Pro) into rostral GABAergic and caudal glutamatergic thalamic neurons (denoted as rTh.N and cTh.N, respectively), or the transition from neurogenesis into gliogenesis at E14.5–16.5 (Figures 1D, 1E, S1C, and S1D).

Of note, we found that these data correctly recapitulated the effect of *SBE1/5* deletions on the expression of *Shh* and Shh target genes. Expression of the basic-helix-loop-helix (bHLH) transcription factor *Olig3* distinguishes thalamic progenitors from other diencephalic cell types.^28^ As expected, differential gene expression analysis in the *Olig3*^+^ progenitor cell subpopulation showed a downregulation of Shh-responsive genes (*Gli1, Ptch1, Nkx2–2*, and *Olig2*) in *ΔSBE1/5* compared with control cells (Figures S1E, S1F, and S1G; Table S1). In addition, a detailed examination of the larger cluster of progenitor cells identified a subgroup with an expression profile consistent with the ZLI (*Shh, Foxa1, Pitx2, Sim2*)^26,40^ (Figure S1H). Differential expression analysis confirmed the strong depletion of *Shh* expression from this subgroup in *ΔSBE1/5* compared with control cells (Figure S1H; Table S2). The persistence of other ZLI markers in mutant cells indicates that the ZLI is not dependent on Shh for the bulk of its formation after E10.0. Altogether, these data represent a unique resource for studies of the developing caudal diencephalon and a substantial increase in cell type resolution with respect to previous single-cell RNA-seq datasets of this brain region.^37,38,41–43^

### Most thalamic nuclei have well-defined molecular identities at E18.5

**Figure 2.**
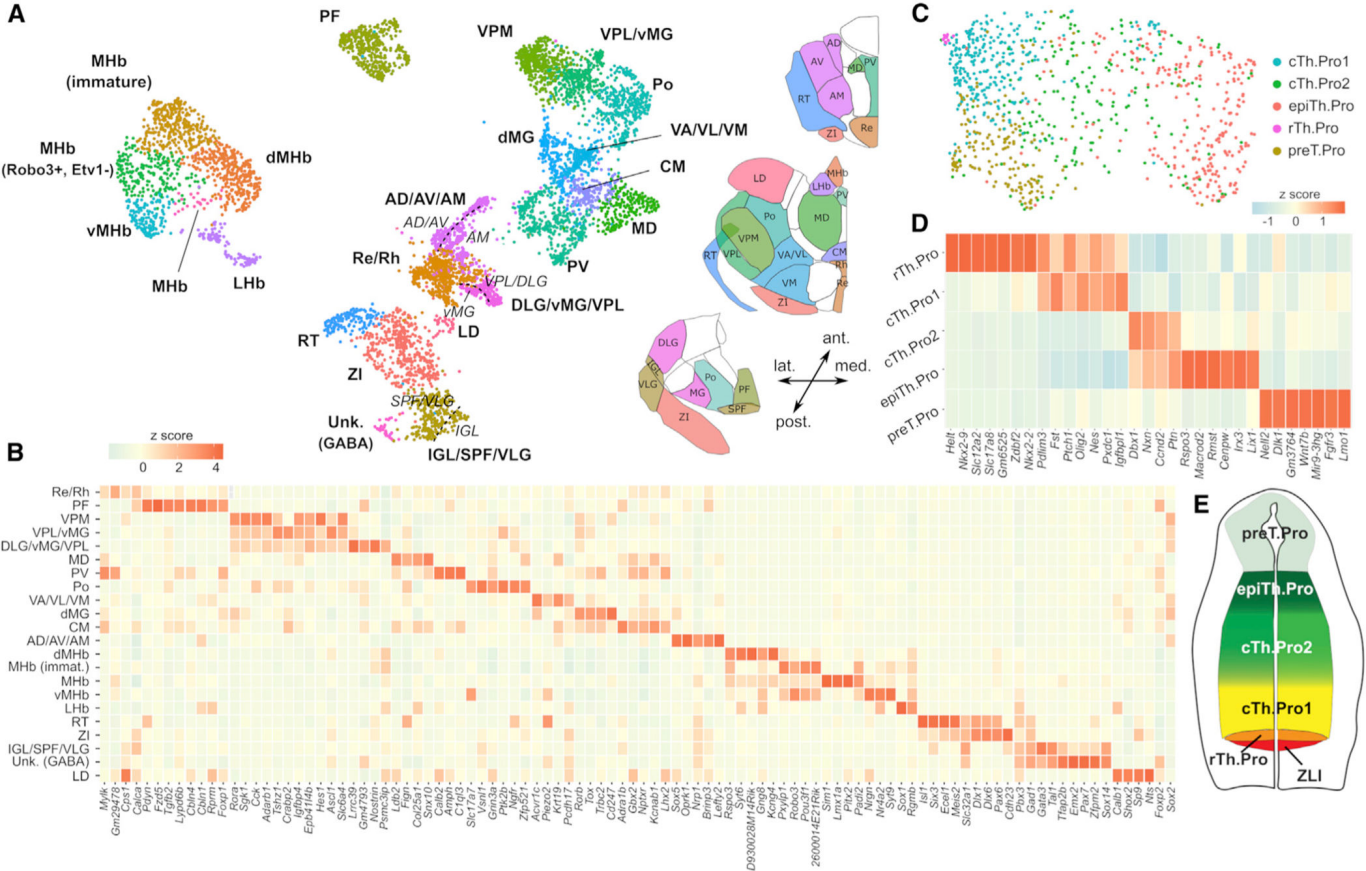
Thalamic nuclei emerge from a diverse pool of progenitor populations and acquire unique transcriptional identities during embryogenesis (A) UMAP representation of the mRNA expression data of E18.5 control cells from the post-mitotic cTh.N, rTh.N, ZI, habenula, and reticular complex cell populations. In total, this analysis identified 23 cell subpopulations with distinct transcriptomic profiles (bold labels). Using a spectral graph method, we split some of these populations into smaller transcriptional identities (italic labels). (B) Heatmap depicting the expression of the top differentially expressed genes in the cell populations from (A). (C) UMAP representation of the mRNA expression data of E12.5 control cells from the *Olig3*^+^ thalamic progenitor population. In total, three thalamic (rTh.Pro, cTh.Pro1, and cTh.Pro2), one epithalamic (epiTh.Pro), and one pretectal (preT.Pro) progenitor populations were identified with distinct transcriptomic profiles. (D) Heatmap depicting the expression of the top differentially expressed genes in the cell populations identified in (C). (E) Schematic showing the rostro-caudal organization of the identified progenitor cell populations in the developing thalamus as inferred from RNA *in situ* hybridization data. See Figure S2; Tables S3 and S4.

Gene expression signatures of distinct thalamic nuclei have been described in adult mice or at single stages of embryonic development, but not in a coordinated manner across developmental time.^2,25,36–38,42^ We sought to systematically characterize the cell populations that define thalamic nuclei throughout embryonic development. For this purpose, we performed a separate clustering and differential gene expression analysis of E18.5 control cells from the cTh.N, rTh.N, zona incerta (ZI), habenula, and reticular complex (RT) post-mitotic cell populations. This analysis identified 23 cell subpopulations with distinct transcriptomic profiles (Figures 2A and 2B; Table S3). By comparing differentially expressed genes across subpopulations with RNA *in situ* hybridization data from the Allen Developing Mouse Brain Atlas,^44^ we were able to assign one or more thalamic nuclei to each of these subpopulations (Figures 2A, S2A, and S2B). Most nuclei in the thalamic, habenular, and reticular complexes were localized to distinct regions of the Uniform Manifold Approximation and Projection (UMAP) representation (Figure 2A). However, in some cases, closely related thalamic nuclei, such as the anterodorsal (AD), anteroventral (AV), and anteromedial (AM), were assigned to the same transcriptomic cell subpopulation. We used a spectral graph method^45^ to dissect the transcriptional heterogeneity within the cell populations identified in our clustering analysis and further resolved the transcriptomic signatures of some of these closely related thalamic nuclei (Figures 2A, S2C, and S2D). We were able to distinguish the transcriptomic profile of the AD/AV thalamic nuclei from that of the AM nucleus, and the profile of the IGL nucleus from that of the VLG and subparafascicular (SPF) nuclei. The identities of other nuclei, such as the distinction between the ventral anterior (VA), ventral lateral (VL), and ventral medial (VM) nuclei, were only partially resolved at this stage of development (Figures S2C and S2D). In other cases, such as for the ventral posterolateral (VPL) and the medial geniculate (MG) thalamic nuclei, we observed multiple transcriptomic subpopulations associated with the same nucleus, suggesting the existence of several cell populations within these nuclei. For example, we identified three distinct transcriptomic populations contributing to the MG nucleus. All of these populations expressed *Gbx2, Lhx2, Rorα,* and *Rorβ*, and demonstrate co-expression of *Sox2* and *Foxp2* in the same cells. However, two of these transcriptomic populations had high expression of ventromedial geniculate (vMG) markers (*Slc6a4, Adarb1, Tshz1, Dlk1*), whereas the other one expressed a dorsomedial geniculate (dMG) marker (*Prox1*).^25^ In addition, the two populations with high expression of vMG markers differed in the expression of multiple genes (Figures 2B and S2A; Table S3), such as *Sp9, Synp2, Nostrin,* and *Psmc3ip*.

Sensory inputs in the brain follow thalamocortical loops, where first-order thalamic nuclei project peripheral sensory inputs onto the primary sensory cortex, and higher-order thalamic nuclei receive their input from the primary sensory cortex and project it back to a secondary cortex.^19,46,47^ The hierarchical position that each nucleus occupies in these circuits has been shown to be the primary determinant of the postnatal transcriptional identity of somatosensory, visual, and auditory thalamic nuclei in postnatal mice.^25^ To assess whether the shared transcriptional programs between same-order nuclei are established earlier during embryogenesis, we projected the postnatal day 3 (P3) differential gene expression signatures of first- and higher-order nuclei from a previous study^25^ onto our single-cell gene expression data of E18.5 thalamic nuclei (Methods S1). This analysis revealed that the postnatal gene expression signatures are already present at E18.5 (Figure S3). Taken together, these data unveil unique molecular signatures that distinguish most thalamic nuclei prior to birth and further suggest that much of the complex structure of the caudal diencephalon is encoded by genetic programs that are active during embryonic development.

### Thalamic progenitors are a heterogeneous cell population

We next characterized the cellular heterogeneity of the *Olig3*^+^ progenitor subpopulation in E12.5 control embryos. Since cell cycle is tightly coupled to cell differentiation during early neurogenesis,^48^ an unsupervised analysis of the progenitor cell population using the most variable genes failed to reveal distinct progenitor types. Regressing out the expression of cell cycle genes did not rectify this issue. To circumvent this problem in the analysis, we adopted a semi-supervised approach in which we compared the transcriptional profile of progenitor cells based on the expression of genes that were highly correlated or anti-correlated with a set of pre-specified markers, including *Nkx2–2, Olig2, Dbx1*, and *Rspo3*. These genes were previously shown to mark distinct and partially overlapping progenitor domains distributed along the rostral to caudal axis of the thalamus.^28,37^

Clustering and differential expression analysis based on this approach identified five transcriptionally distinct populations of progenitors (Figures 2C and 2D; Table S4). We named these progenitor populations as rTh.Pro (rostral thalamic progenitor), cTh.Pro1 (caudal thalamic progenitor 1), cTh.Pro2 (caudal thalamic progenitor 2), epiTh.Pro (epithalamic progenitor), and preT.Pro (pretectal progenitor) based on their rostro-caudal order in the neural tube (Figure 2E). The two most rostral thalamic progenitor populations (rTh.Pro and cTh.Pro1) were characterized by the expression of *Shh*-responsive genes, such as *Nkx2–2, Olig2,* and *Ptch1* (Figure 2D), consistent with previous findings.^12^ High levels of expression of Shh-responsive genes were excluded from cTh.Pro2, epiTh.Pro, and preT.Pro domains, which were defined by distinct sets of differentially expressed transcripts (Figure 2D). EpiTh.Pro progenitors were characterized by the expression of markers, such as *Macrod2* and *Rspo3*, that continued to be expressed in neurons with an epithalamic identity across all differentiation stages, including neurons of the habenula (Figure S4). These markers were upregulated in distinct but partially overlapping subpopulations of the epiTh.Pro domain, suggesting the presence of additional heterogeneity within these progenitors. EpiTh.Pro progenitors had lower but non-vanishing expression of *Olig3* compared with other thalamic progenitors (Figure S4A). This result is consistent with a gradual transition between thalamic progenitor domains and *Olig3*^−^ habenular and pretectal progenitors. The most caudal progenitor subpopulation (preT.Pro) was characterized by the expression of pretectal markers (*Pax3, Meis1, Lmo1*) (Table S4). On the basis of these results, we hypothesize that different cell lineages derived from distinct thalamic progenitor populations give rise to the diversity of thalamic nuclei. Our results also suggest the presence of soft boundaries between progenitor domains, where some cells co-express markers from multiple progenitor domains.

### Glutamatergic thalamic nuclei emerge sequentially through the coordinated action of three distinct cell lineages

**Figure 3.**
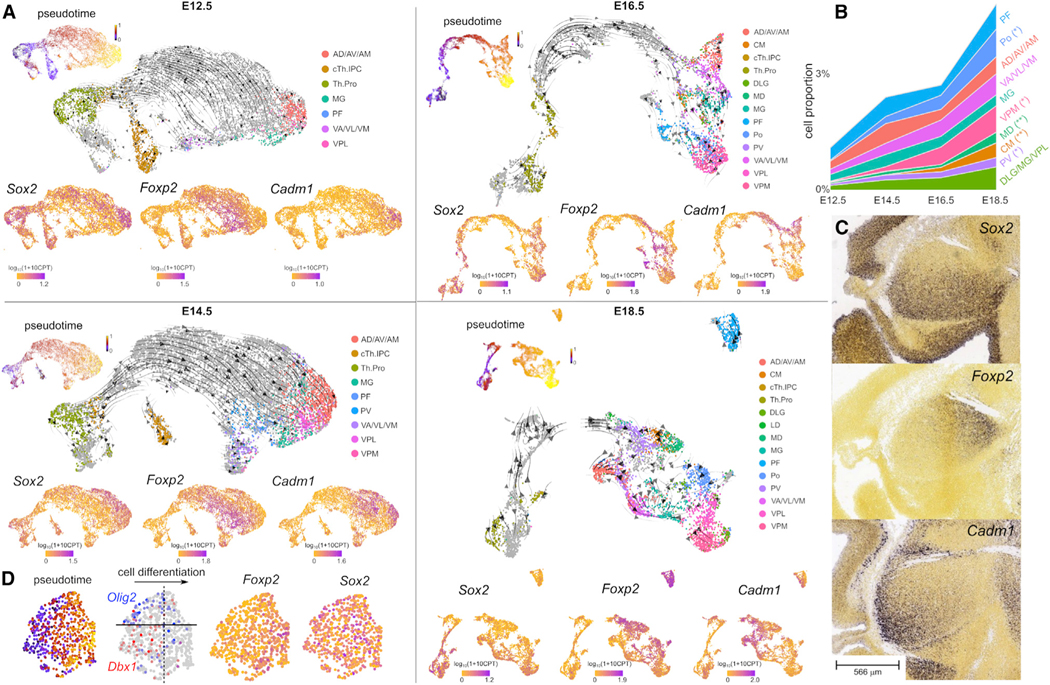
Glutamatergic thalamic cell lineages regionalize the thalamus into distinct molecular domains (A) UMAP representation and RNA velocity field of the single-cell RNA-seq data of the glutamatergic cell lineage in control embryos at each developmental stage. For reference, the same UMAP is also colored by the inferred cell differentiation pseudo-time and the gene expression levels of *Sox2, Foxp2,* and *Cadm1*. The expression of *Sox2* and *Foxp2* genes separates differentiation trajectories into Sox2^+^ Foxp2^−^ (cTh.N1) and *Sox2*^−^ *Foxp2*^+^ (cTh.N2) populations. (B) Proportion of cells belonging to different glutamatergic thalamic nuclei at each developmental stage. Distinct thalamic subpopulations emerge at different time points. Late- and intermediate-emerging cell subpopulations are indicated by double asterisk (**) and single asterisk (*), respectively. (C) Whole-mount RNA *in situ* hybridization for *Sox2, Foxp2,* and *Cadm1* in sagittal sections of the E13.5 diencephalon. The expression of these markers regionalizes the developing thalamus into distinct domains. Image credit: Allen Institute. (D) Glutamatergic thalamic IPCs are a heterogeneous population of cells with contributions from both cTh.Pro1 and cTh.Pro2 progenitors. The UMAP representation of the single-cell RNA-seq data corresponding to the glutamatergic thalamic IPC population from E14.5 control embryos is colored by the inferred cell differentiation pseudo-time and the gene expression levels of *Olig2, Dbx1, Foxp2,* and *Sox2*. CPT, counts per thousand. See Figure S5; Tables S4 and S5.

To uncover the transcriptomic lineages that relate thalamic progenitors at E12.5 to post-mitotic thalamic neurons at E18.5, we devised a semi-supervised approach for transferring the E12.5 and E18.5 cell annotations across all time points and identified cells with a similar gene expression profile (STAR Methods). We disaggregated the progenitor and post-mitotic populations into smaller clusters in the full single-cell RNA-seq representation and used the E12.5 and E18.5 cell annotations to label each cluster. We then constructed separate single-cell gene expression representations for glutamatergic cell lineages (*Olig3*^+^ cTh.Pro, cTh.N early post-mitotic, and cTh.N clusters) and computed the RNA velocity vector field^49,50^ in these representations (Figure 3A).

Each embryonic day was analyzed independently, since neurodevelopmental lineages may change across time as a consequence of shifts in the progenitor identities. As expected, the inferred differentiation trajectories connected the progenitors with the post-mitotic neurons in each developmental stage (Figure 3A). The differentiation trajectories included a population of intermediate progenitor cells (IPCs), characterized by the co-expression of neurogenic determinants (*Neurog1, Neurog2*) and mitotic markers (*Top2a, Mki67*).^51^ The number of *Olig3*^+^ progenitors and IPCs rapidly diminished after E14.5 due to the transition into gliogenesis, while post-mitotic cells continued to differentiate well beyond E14.5.

Our analysis revealed the sequential emergence of glutamatergic thalamic nuclei, where lateral and intermediate nuclei such as the AD/AV/AM, dorsolateral geniculate (DLG), MG, VPL, parafascicular (PF), and posterior (Po) nuclei differentiate first, and medial nuclei such as the centromedian (CM), MD, and paraventricular (PV) differentiate later (Figures 3A and 3B). Analysis of gene expression along the differentiation trajectories showed that the expression of *Sox2* and *Foxp2* was anti-correlated with each other (Spearman’s correlation coefficient *r* = − 0.2, p < 2 × 10^−16^) and separated glutamatergic cell differentiation trajectories into *Sox2*^+^ *Foxp2*^−^ and *Sox2*^−^ *Foxp2*^+^ trajectories at all developmental stages (Figure 3A). These results suggest the presence of two major cell differentiation lineages of glutamatergic thalamic neurons, which we denote as cTh.N1 (caudal thalamic neuron 1) and cTh.N2 (caudal thalamic neuron 2). Differential gene expression analysis at E12.5 revealed 148 differentially expressed genes between cTh.N1 and cTh.N2 cell populations with a fold change >2 (Table S5, adjusted p value <0.05).

To understand the relationship between these putative cell differentiation lineages and the observed diversity of thalamic nuclei and progenitors, we examined the expression of *Sox2* and *Foxp2* in these populations. The expression of *Sox2* was ubiquitous in the progenitors and was preserved throughout the cTh.N1 population, including in the differentiated ventroposterior medial (VPM), AD/AV, AM, and VA/VL/VM nuclei (Figures 3A and S5A). By contrast, the expression of *Foxp2* was initiated in a subset of IPCs (Figure 3D) and preserved throughout the cTh.N2 population, including the PF, Po, PV, MD, and CM nuclei (Figures 3A and S5A). Importantly, these results establish a basis for the distinction between *Sox2*− and *Foxp2*-expressing thalamic nuclei observed in E18.5 cells (Figure 2B, far right columns).

Our analysis also revealed a separation of the cTh.N1 differentiation trajectory into *Cadm1*^+^ and *Cadm1*^−^ subpopulations (Figure 3A). The expression of *Cadm1* was initiated in a subset of early cTh.N1 post-mitotic cells and preserved in the thalamic nuclei that derive from this population, including the AD/AV, AM, VA/VL, and VM nuclei (Figures 3A and S5A). RNA *in situ* hybridization confirmed the regionalization of the thalamus into three distinct domains based on the expression of *Sox2, Foxp2,* and *Cadm1,* with *Sox2* being expressed rostrally and *Foxp2* caudally (Figure 3C). Thus, our analysis of differentiation trajectories in the single-cell data is consistent with the emergence of glutamatergic thalamic nuclei from distinct transcriptomic lineages marked by the expression of *Sox2* and *Foxp2*. The rostro-caudal organization of these transcriptomic populations together with the organization of thalamic progenitors in the neural tube suggests that these lineages originate from distinct subpopulations of glutamatergic progenitors. A similar analysis of the GABAergic cell populations showed that GABAergic neurons of the thalamus derive from *Tal1*^+^ neural progenitors and are characterized by the expression of Shh-responsive genes (Methods S2 and Figure S6).

### Glutamatergic thalamic IPCs are a heterogeneous population of cells with contributions from both cTh.Pro1 and cTh.Pro2 progenitors

Despite our ability to transfer annotations across time points, a gene expression bottleneck within IPCs prevented us from relating the aforementioned transcriptomic cell trajectories to individual thalamic progenitor identities. Most of the genes expressed in progenitor cells ceased to be expressed after mitotic arrest, except for *Hs3st1*, which marked both the cTh.Pro1 and cTh.N1 populations (Figure S5B). However, a closer look at the IPC population and its RNA velocity field revealed the presence of two subsets of early IPCs, characterized by the expression of the cTh.Pro1 marker *Olig2*, and the cTh.Pro2 marker *Dbx1*, as well as two subsets of late IPCs, respectively, characterized by the presence and absence of *Foxp2* expression (Figure 3D). The *Foxp2*^+^ subpopulation of IPCs appeared contiguous in the gene expression space to the *Dbx1*^+^ subpopulation, whereas the *Foxp2*^−^ subpopulation appeared contiguous to the *Olig2*^+^ subpopulation. We did not observe the expression of epiTh.Pro markers in the IPC cluster, indicating that the formation of habenular nuclei does not involve the generation of IPCs from these progenitors. These results suggest that the cTh.N1 and cTh.N2 cell populations are respectively derived from cTh.Pro1 and cTh.Pro2 progenitors, and that both lineages expand through the generation of distinct IPCs.

### Shh signaling is required for rTh.Pro and cTh.Pro1 progenitor specification and expansion

**Figure 4.**
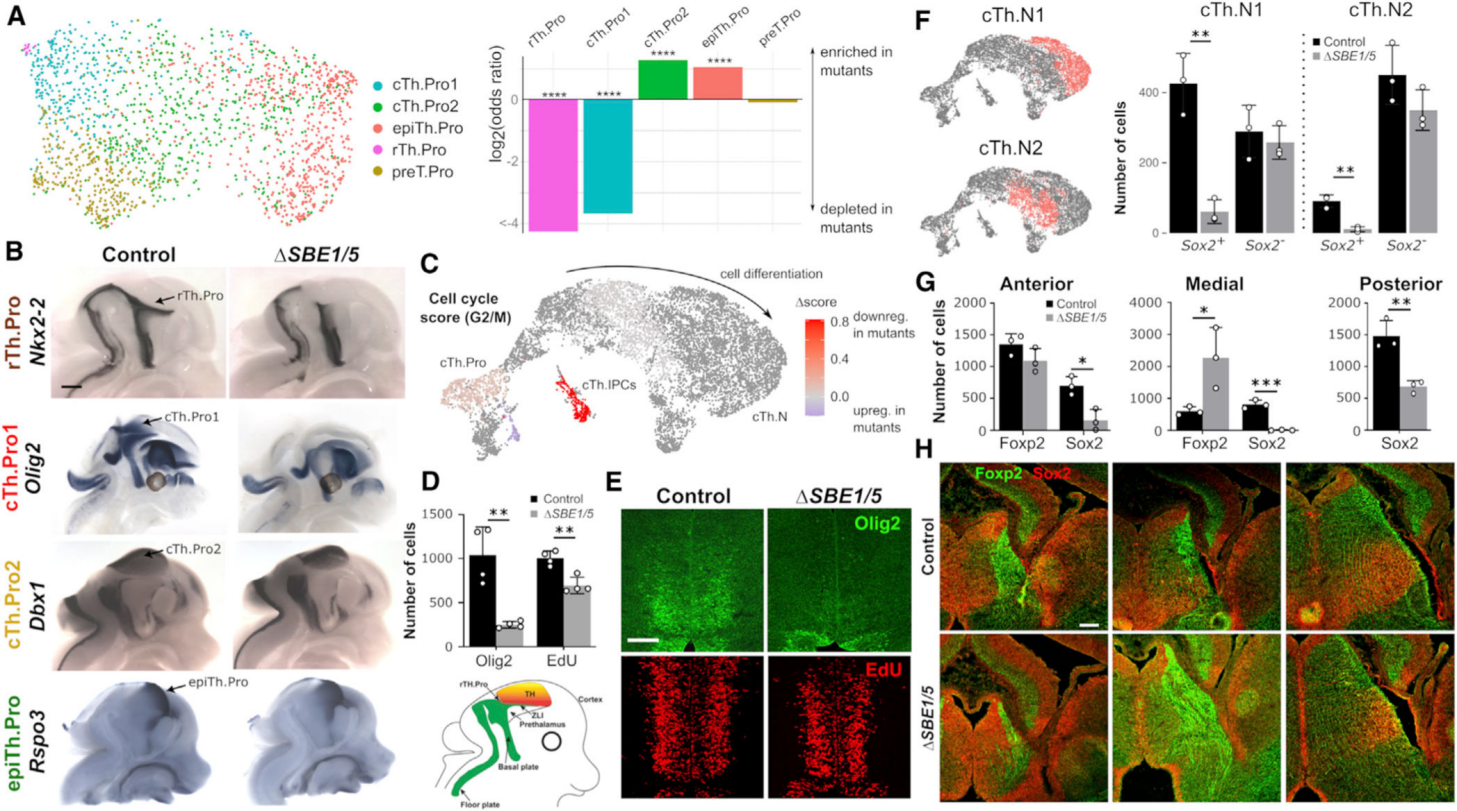
Shh signaling is required for the specification and expansion of rTh.Pro and cTh.Pro1 thalamic progenitors (A) UMAP representation of the mRNA expression data of E12.5 control and *ΔSBE1/5* cells from the *Olig3*^+^ thalamic progenitor population (left). The bar plot shows the depletion or enrichment of cells between control and *ΔSBE1/5* embryos in each progenitor cluster, indicating a strong depletion of rTh.Pro and cTh.Pro1 cells in *ΔSBE1/5* embryos (****p < 0.0001, two-sided Fisher’s exact test, n = 1,789 cells). (B) Whole-mount RNA *in situ* hybridization for *Nkx2–2, Olig2, Dbx1,* and *Rspo3* in E12.5 control and *ΔSBE1/5* embryos, confirming the strong depletion of rTh.Pro and cTh.Pro1 cells in mutant embryos (n = 3). Scale bar, 500 μm. (C) UMAP representation of the glutamatergic thalamic cell lineage at E14.5 colored by the difference in the G2/M cell cycle gene expression score between control and *ΔSBE1/5* cells. The analysis shows G2/M cell cycle genes are downregulated in thalamic progenitors and IPCs in *ΔSBE1/5* embryos. (D and E) EdU incorporation and immunofluorescence staining for Olig2 on coronal sections of the thalamus in control and *ΔSBE1/5* E13.5 embryos, showing a depletion of Olig2-expressing cells (cTh.Pro1 cells) and a reduction of cell cycle in IPCs (**p < 0.01, two-sided t test, n = 4 mice of each condition; error bars represent standard deviation). Scale bar, 100 μm. (F) The total number of *Sox2*-expressing cells in the post-mitotic cTh.N1 and cTh.N2 transcriptomic cell lineages is largely depleted in *ΔSBE1/5* compared with control embryos at E14.5. For reference, the location of the post-mitotic cTh.N1 and cTh.N2 clusters in the UMAP representation of the glutamatergic thalamic cell lineage is shown on the left (**p < 0.01, two-sided t test, n = 3 mice; error bars represent standard deviation). (G and H) Immunofluorescence staining for Sox2 and Foxp2 in anterior, medial, and posterior sections of E14.5 control and *ΔSBE1/5* embryos. A depletion of Sox2^+^ cells and an expansion of Foxp2^+^ cells is observed in mutant embryos (*p < 0.05, **p < 0.01, ***p < 0.001, two-sided t test, n = 3 mice of each condition; error bars represent standard deviation). Scale bar, 100 μm. See Figure S7.

The inference of developmental trajectories from unperturbed single-cell transcriptomic data has proved to be misleading in some situations.^52^ Therefore, we studied the effect of the *ΔSBE1/5* deletions on the transcriptomic cell populations to further test our model of thalamic development. To determine the effect of Shh signaling on thalamic progenitor specification, we examined the differences between *Olig3*^+^ progenitors in control and *ΔSBE1/5* embryos at E12.5. Our analysis of the single-cell RNA-seq data identified a strong depletion of rTh.Pro and cTh.Pro1 progenitors in *ΔSBE1/5* embryos (Figure 4A, odds ratio = 13.5, Fisher’s exact test p < 10^−10^), consistent with previous studies demonstrating that the specification of these two progenitor identities is dependent on Shh signaling.^12,13^ RNA *in situ* hybridization for *Nkx2–2* and *Olig2* confirmed the strong depletion of rTh.Pro and cTh.Pro1 progenitors, respectively, in *ΔSBE1/5* embryos (Figure 4B). Our analysis of the single-cell RNA-seq data also identified a moderate expansion of the cTh.Pro2 progenitor pool in mutant embryos (Figure 4A, odds ratio = 0.23, Fisher’s exact test p < 10^−10^). However, we were unable to confirm this expansion with *in situ* data (Figure 4B). Differential gene expression analysis between control and *ΔSBE1/5 Olig3*^+^ glutamatergic progenitor cells revealed the downregulation of cell cycle genes in mutant cells (Figures 4C and S7A; Table S1). This effect was particularly prominent in the IPC population (Figure 4C). We confirmed the observed downregulation of cell cycle in *ΔSBE1/5* cTh.Pro1 progenitors by means of a 5-ethynyl-2′-deoxyuridine (EdU) incorporation assay (Figures 4D, 4E, and S7B). These results suggest that the specification and expansion of cTh.Pro1 progenitors is dependent on Shh. Moreover, consistent with our hypothesis that cTh.N1 cells derive from cTh.Pro1 progenitors, we observed that the reduction in cTh.Pro1 progenitors in *ΔSBE1/5* mice was accompanied by a substantial reduction in the number of *Sox2*^+^ post-mitotic cells in the glutamatergic cTh.N1 transcriptomic lineage in our single-cell RNA-seq data and a higher proportion of *Foxp2* expressing cells in this lineage (Figures 4F and S8A). These results were confirmed by immunofluorescence staining for Sox2 and Foxp2 in control and *ΔSBE1/5* embryos at E14.5 (Figures 4G and 4H). We conclude that Shh signaling is required for the specification and expansion of rTh.Pro- and cTh.Pro1-derived thalamic cell populations.

### *Nkx2–2* and *Sox2* expressing thalamic nuclei are reduced in *ΔSBE1/5* mice

**Figure 5.**
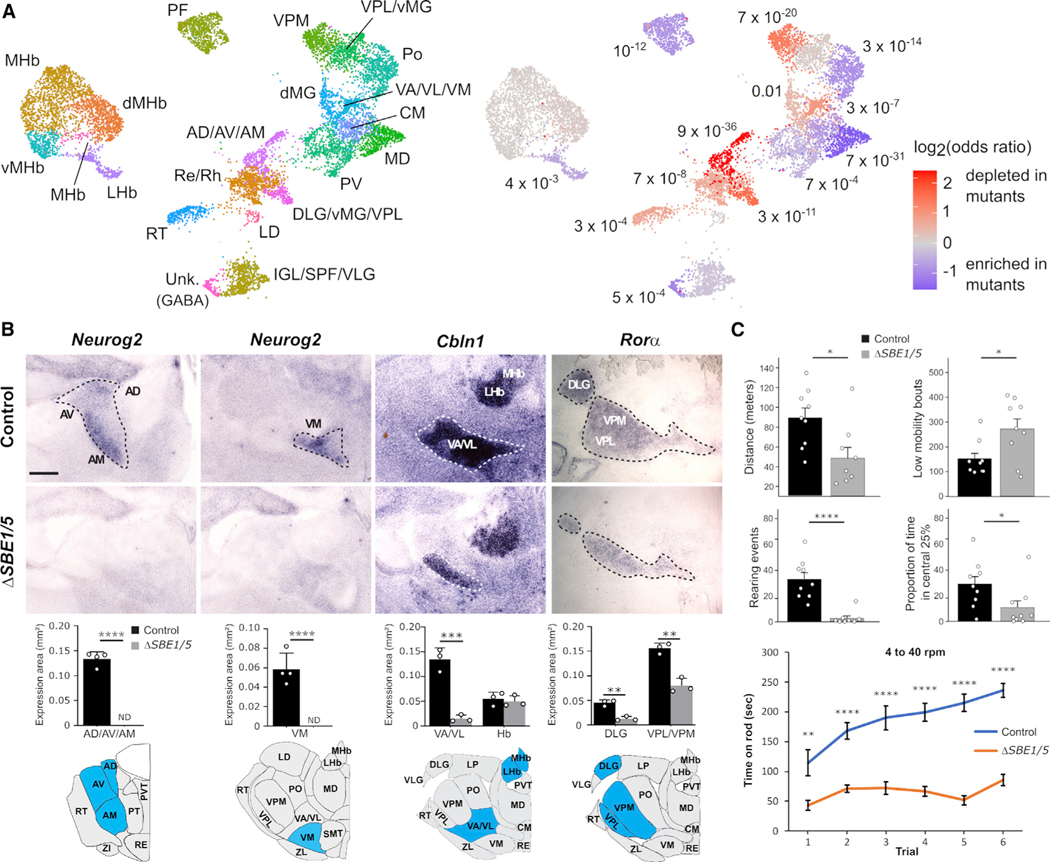
Shh-dependent thalamic nuclei fail to develop in *ΔSBE1/5* mice (A) UMAP representation of the single-cell RNA-seq expression data from control and *ΔSBE1/5* embryos at E18.5 corresponding to post-mitotic cTh.N, rTh.N, ZI, habenula, and reticular complex cell populations (left). The UMAP is colored by the amount of depletion or enrichment in the number of cells of each progenitor subpopulation between control and *ΔSBE1/5* embryos (right). (B) RNA *in situ* hybridization for *Neurog2, Cbln1,* and *Rorα* on coronal sections through the thalamus of control and *ΔSBE1/5* mice at P0 showing reduced expression in AV/AM/AD, VA/VL/VM, DLG, and VPM/VPL thalamic nuclei, consistent with the results of the single-cell RNA-seq analysis (**p < 0.01, ***p < 0.001, ****p < 0.0001, two-sided t test; n = 3 mice of each condition for *Cbln1* and *Rorα*; n = 4 mice of each condition for *Neurog2*; error bars represent standard error of the mean). (C) Locomotor behavior is compromised in adult *ΔSBE1/5* mice as determined by force plate actometer (top four graphs) and rotarod (bottom) assays (*p < 0.05, **p < 0.01, ****p < 0.0001, two-sided t test; n = 8–9 mice of each condition for force plate; n = 10 mice of each condition for rotarod; error bars represent standard error of the mean). See Figure S8.

Based on our model of thalamic development, the observed depletion of cTh.Pro1 progenitors in *ΔSBE1/5* embryos should lead to a failure in the development of Sox2-expressing cTh.N1 thalamic nuclei. Our analysis of single-cell RNA-seq data from post-mitotic glutamatergic cells in control and *ΔSBE1/5* embryos at E18.5 confirmed a large depletion of cells in thalamic nuclei expressing high levels of *Sox2*, including the AD/AV/AM, VA/VL/VM, DLG/MG/VPL, and VPM nuclei (Figure 5A). Consistent with these results, RNA *in situ* hybridization for some of the markers identified in our differential gene expression analysis of these nuclei (Figure 2B) showed a large reduction in staining in *ΔSBE1/5* newborn (P0) mice (Figures 5B and S8B). In particular, markers of the AD/AV/AM and VM nuclei were completely absent in mutant mice, whereas markers of the VA/VL, DLG/MG/VPL, and VPM were largely reduced (Figures 5B and S8B). Additionally, Nkx2–2 immunostaining was absent in the IGL/VLG nuclei in *ΔSBE1/5* mice at P0 (Figure S8B), consistent with the depletion of GABAergic rTh.Pro progenitors (Figure 4A). Taken together, the analysis of single-cell RNA-seq and *in situ* expression data is consistent with a model of thalamic development where glutamatergic *Sox2*-expressing nuclei (cTh.N1 cells) are derived from Shh-dependent cTh.Pro1 progenitors, glutamatergic *Foxp2*-expressing nuclei (cTh.N2 cells) are derived from Shh-independent cTh.Pro2 progenitors, and GABAergic *Nkx2–2*-expressing nuclei (rTh.N cells) are derived from *Shh*-dependent rTh.Pro progenitors.

### Locomotor deficits in *ΔSBE1/5* mice

We next assessed the consequences that abnormal thalamic development might have on animal behavior in *ΔSBE1/5* mutant mice. *ΔSBE1/5* mice are viable and exhibit reduced locomotor activity and rearing in the open field, as well as a significantly impaired initial coordination and subsequent motor learning on the accelerating rotarod test (Figure 5C). These early-onset motor deficits resemble several cardinal features of infantile Parkinson’s disease, including bradykinesia, abnormal gait, and poor coordination (Figure 5C).^53^

The nigrostriatal pathway is a critical component of the basal ganglia motor circuit that degenerates in individuals with Parkinson’s disease. However, unlike other mouse models of the infantile form of this condition that result from tyrosine hydroxylase deficiency,^53^ the rate-limiting enzyme in dopamine synthesis, we observed no defects in the development or maintenance of dopaminergic neurons in the substantia nigra pars compacta or their projections to the striatum in *ΔSBE1/5* mice (Figures S8C and S8D). This result is particularly relevant since SBE1 and SBE5 also regulate *Shh* expression in the ventral midbrain (Figure 1A), which is the source of midbrain dopaminergic neurons. However, since these neurons develop properly in *ΔSBE1/5* mice, likely due to their dependency on an earlier source of Shh, we suspect that another component of the basal ganglia motor circuit is compromised in *ΔSBE1/5* mice.

As described above, the failure to specify sufficient numbers of cTh.Pro1 progenitors in the absence of Shh results in a reduction in the cTh.N1 lineage, which consequently disrupts the formation of motor thalamic nuclei (VA/VL, VM). The deficits in motor thalamic nuclei observed in *ΔSBE1/5* embryos and newborn pups extends into adulthood (Figure S8E). Lesions to VA/VL are known to cause severe motor dysfunction in a variety of animal models.^54–57^ Therefore, the most parsimonious explanation for the motor impairment phenotype in *ΔSBE1/5* mice is the neurodevelopmental defect in motor thalamic nuclei formation, although secondary effects in other motor circuits cannot be ruled out.

## DISCUSSION

### Single-cell trajectories support the “outside-in” model of thalamic neurogenesis

Our study provides a comprehensive transcriptome-wide analysis of thalamic progenitors and their trajectories into thalamic nuclei during embryonic stages of brain development. We demonstrate that molecular signatures of thalamic neuronal subtypes can be readily distinguished as early as E12.5, prior to their aggregation into histologically distinct thalamic nuclei.^26^ These data lend support to the outside-in model of thalamic neurogenesis, whereby early-born neurons contribute to lateral thalamic nuclei and later-born neurons contribute to medial thalamic nuclei.^36,58^ Our work further extends this model with molecular insights into the mechanisms by which diverse thalamic nuclei acquire their identities. By following single-cell trajectories over developmental time, our data are consistent with a model in which thalamic nuclei arise from three distinct cellular lineages. In this model, Shh-responsive cTh.Pro1 progenitors give rise to glutamatergic neurons (cTh.N1) within sensory (DLG, VPM/VPL, vMG), motor (VA/VL, VM), and anterior (AD, AV, AM) nuclei that occupy predominantly ventrolateral regions of the thalamus. Shh-responsive rTh.Pro progenitors give rise to GABAergic neurons (rTh.N) in VLG and IGL thalamic nuclei, in agreement with previous findings.^12,13^ In contrast, Shh-independent cTh.Pro2 progenitors give rise to neurons (cTh.N2) within nuclei (e.g., PV, MD, CM) that form at more dorsomedial positions of the thalamus.

**Figure 6.**
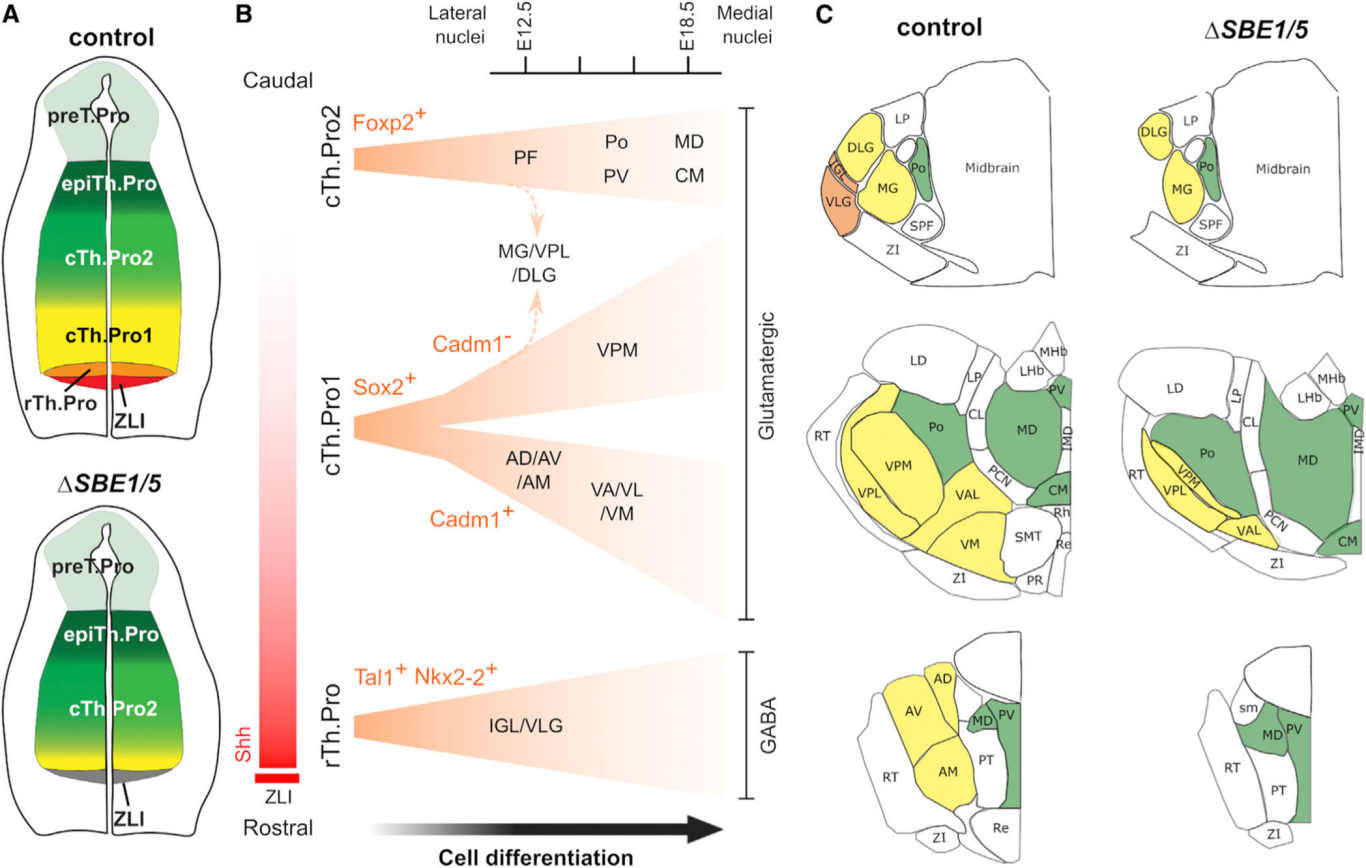
Model of thalamic development (A) Thalamic progenitors are organized rostro-caudally in the neural tube and acquire distinct identities based on their exposure to the Shh morphogen gradient originating from the ZLI and basal plate (top). In *ΔSBE1/5* mutants, the pool of rTh.Pro and cTh.Pro1 progenitors are not specified and fail to expand due to the lack of Shh signaling (bottom). (B) rTh.Pro, cTh.Pro1, and cTh.Pro2 give rise to different cell lineages, which are characterized, respectively, by the expression of *Tal1*, *Sox2*, and *Foxp2* at all post-mitotic stages of cell differentiation. The *Sox2*^+^ lineage is divided into two sub-lineages soon after post-mitotic arrest of the progenitors. These sub-lineages can be distinguished by the expression of *Cadm1*. Different cell lineages contribute to different thalamic nuclei in a sequential temporal manner, where lateral nuclei are formed first and medial nuclei are formed later. An exception to this model is the MG/VPL/DLG, which receives contributions from both cTh.Pro1 and cTh.Pro2 progenitors. (C) Thalamic nuclei derived from Shh-dependent (rTh.Pro and cTh.Pro1) progenitors (labeled in orange and yellow, respectively) are either absent, or significantly reduced, in *ΔSBE1/5* mice, whereas nuclei derived from cTh.Pro2 progenitors (labeled in green) are partially expanded in the absence of Shh.

The bifurcation of transcriptomic trajectories according to thalamic progenitor identity is in general agreement with results from *in vivo* clonal analyses.^36,59^ These studies revealed that individual thalamic progenitors give rise to many neurons that populate multiple thalamic nuclei. They also demonstrated that the clonal relationship between thalamic nuclei is determined primarily by the rostro-caudal and dorsoventral positions of thalamic progenitors. These findings are consistent with our observations that thalamic nuclei originate from a small number of spatially and temporally segregated neural progenitors located along the primary axes of the developing thalamus (Figure 6A).

### IPCs mediate the transition between thalamic progenitors and neurons

Previous work described the presence of IPCs in the thalamus but not their specific lineage relationships with thalamic progenitors and neurons.^37,51^ Our data are consistent with Olig2^+^ Sox2^+^ IPCs giving rise to ventrolateral thalamic nuclei and Foxp2^+^ IPCs giving rise to dorsomedial thalamic nuclei (Figures 6B and 6C). Intermixing of Sox2^+^ and Foxp2^+^ populations was observed in a subset of sensory nuclei (Figures 6B and 6C). The Sox2^+^ transcriptomic trajectory is further partitioned by *Cadm1* expression. In addition to marking distinct and overlapping thalamic IPCs and neurons, Sox2 and Foxp2 are required to regulate functional properties of their respective thalamic lineages.^60,61^ In particular, the cell-autonomous loss of VP (VPM/VPL), PF, and Po nuclei in *Foxp2* mutants at E14.5^60^ suggests that Foxp2 may be required for the transition of IPCs to a subset of cTh.N2 thalamic neurons. It is worth noting that Foxp2 has a similar role in the cerebral cortex, where it regulates the formation of IPCs and their transition to cortical neurons.^62^ Less is known about the role of Sox2 in thalamic neurogenesis. However, the conditional knockout of Sox2 in post-mitotic thalamic neurons results in a reduction in the size and connectivity of sensory nuclei.^61^ Thus, the developmental segregation of cTh.Pro, IPC, and cTh.N subtypes by lineage-determining transcription factors likely plays an important role in the formation of distinct thalamic nuclei.

### Impaired locomotor activity in *ΔSBE1/5* mutant mice

Deciphering the pathogenic mechanisms of thalamic dysfunction in *ΔSBE1/5* mutants provided novel insight into the etiology of a neurodevelopmental movement disorder with a similar phenotype to infantile Parkinson’s disease. We have shown that the spatiotemporal regulation of *Shh* expression in the ZLI and basal plate of the caudal diencephalon is critical for the elaboration of thalamic progenitor identities that populate multiple thalamic nuclei, including the motor thalamus (VA/VL, VM), principal sensory nuclei (DLG, VPM/VPL, vMG), and anterior thalamic nuclei (AD, AV, AM) (Figures 5A and 5B). It would therefore appear that the timing of Shh depletion may explain many of the unique features of our mouse model compared with other conditional *Shh* mutants in this brain region.^11,12^

We propose that the motor impairment in *ΔSBE1/5* mutant mice is attributed to loss of the Shh-dependent cTh.Pro1 subtype of thalamic progenitors, resulting in a reduced number of Olig2^+^ Sox2^+^ IPCs and, subsequently, fewer cTh.N1 thalamic neurons populating motor thalamic nuclei (VA/VL, VM) during embryonic development. Future studies will address the consequences that alterations of other thalamic nuclei have for sensory, motor, and other behaviors in *ΔSBE1/5* mutant mice. These experiments have the potential to further improve our basic understanding of thalamic development, which has consistently lagged behind other brain regions, and may also provide novel insights into the etiology of other circuit-level endophenotypes associated with abnormal motor and sensory information processing that occur in a variety of neurodevelopmental disorders.^63–66^

### Limitations of the study

Our study maps the transcriptomic trajectories and Shh dependencies of thalamic progenitors into specific thalamic nuclei. However, since not all thalamic nuclei were readily discerned by our single-cell methods at E18.5, it is likely that their differentiation continues postnatally, beyond the scope of our analysis. Complementary lineage tracing experiments will be useful to confirm and potentially refine some of the sub-lineages presented in this paper. Moreover, elucidating the cell-intrinsic mechanisms by which thalamic nuclei acquire their unique transcriptional identities will require follow-up experiments integrating functional and multi-omics approaches (e.g., assay for transposase-accessible chromatin with high-throughput sequencing [ATAC-seq], chromatin immunoprecipitation sequencing [ChIP-seq]). Finally, despite our determination that Shh instructs cTh.Pro1 and rTh.Pro progenitors to adopt specific trajectories, the extrinsic signals responsible for patterning cTh.Pro2 and epiTh.Pro remain to be confirmed.

## STAR★METHODS

### RESOURCE AVAILABILITY

#### Lead contact

Further information and requests for resources and reagents should be directed to and will be fulfilled by the lead contact, Pablo G. Camara (pcamara@pennmedicine.upenn.edu).

#### Materials availability

This study has generated mouse lines, which are listed in the key resource table. Mouse lines are available upon request.

#### Data and code availability

- Single-cell RNA-seq data have been deposited at Short Read Archive (SRA), Gene Omnibus Archive (GEO) (GSE211701), Mendeley Data, and cellxgene and are publicly available as the date of publication. Accession numbers are listed in the key resources table.
- This paper does not report original code.
- Any additional information required to reanalyze the data reported in this paper is available from the lead contact upon request.

### EXPERIMENTAL MODEL AND SUBJECT DETAILS

All mouse experiments were performed in accordance with the ethical guidelines of the National Institutes of Health and with the approval of the Institutional Animal Care and Use Committee of the University of Pennsylvania. Mice were housed in Thoren caging units under a constant 12-h light/dark cycle. Mice carrying targeted deletions of SBE1 and SBE5 were described previously.^13,39^ To generate *Shh*^*ΔSBE1ΔSBE5/ΔSBE1ΔSBE5*^ double homozygous mutant embryos, the *Shh*^*ΔSBE5/*+^ line was first crossed with *Shh*^*ΔSBE1/ΔSBE1*^ mutants. *Shh*^*ΔSBE1/+; ΔSBE5/+*^ males, carrying the SBE1 and SBE5 deletions in *trans*, were then bred to wild type CD1 females. The progeny from this cross were screened for recombination events that placed the SBE1 and SBE5 deletions in cis (1/600 offspring). The *Shh*^*ΔSBE1ΔSBE5/+*^ double heterozygous animals were then intercrossed to generate *Shh*^*ΔSBE1ΔSBE5/ΔSBE1ΔSBE5*^ double homozygous mice and embryos. The 429M20eGFP BAC transgenic mouse reporter line (referred to herein as *Shh-GFP*) expresses eGFP in the ZLI and basal plate of the caudal diencephalon under the transcriptional control of SBE1, as described previously.^67^ Cbln2-mVenus mice were described previously.^68^ Both male and female mice (3–5 months of age) were used in behavioral studies. No sex specific differences were observed between genotypes.

### METHOD DETAILS

#### *In situ* hybridization

Embryonic or neonatal (P0) brains were collected from timed pregnant females (vaginal plug = E0.5). For whole-mount RNA *in situ* hybridization, heads were fixed in 4% paraformaldehyde at 4°C for overnight, bisected along the mid-sagittal plane and hybridized with digoxygenin-UTP-labeled riboprobes as previously described.^67^ For RNA *in situ* hybridization on sections, heads were dissected and fixed for 2 h in 4% paraformaldehyde at 4°C, then washed in PBS. Samples were cryoprotected overnight in 30% sucrose/PBS then snap frozen in OCT embedding compound (Sakura Finetek Torrence, CA). Samples were serially sectioned along the coronal plane at 16 μm (for E12.5 and E13.5 embryos), 18 μm (for E14.5 embryos) or 20 μm (for E18.5 embryos) thickness using a cryostat (Leica Biosystems, CM3050 S). Sections were hybridized with digoxigenin- UTP-labeled riboprobes as previously described.^72^

#### Immunohistochemistry

Brains were processed for immunohistochemistry in the same fashion as for *in situ* hybridization on sections. Brain sections were stained with DAPI and incubated with the following primary antibodies: rabbit anti-Foxp2 (1:200, Abcam, ab16046), mouse anti-Nkx2.2 (1:300, DSHB, 74.5A), rabbit anti-Olig2 (1:300, Millipore, AB9610), mouse anti-Sox2 (1:100, R&D Systems, MAB2018), rabbit anti-Sox2 (1:300, Millipore, Cat#AB5603), and rabbit anti-Tyrosine Hydroxylase (1:1000, Pel-Freez, P40101–0). Detection of primary antibodies was achieved using secondary antibodies conjugated to goat anti-mouse Alexa 488 (1:400, Thermo Fisher, Cat#A28175), goat anti-rabbit Alexa 488(1:400, Thermo Fisher, Cat#A-11008), and goat anti-rabbit Alexa 594 (1:400, Thermo Fisher, Cat#A-11037). Specimens were imaged on a Leica TCS SP8 MP system.

#### EdU incorporation

EdU was dissolved in sterile water and administered to pregnant dams via intraperitoneal injection at a concentration of 50 μg/g of body weight, 2 h prior to embryo harvest (Molecular Probes). Embryos were fixed in 4% paraformaldehyde at 4°C for 2 h, then were processed in the same fashion as for *in situ* hybridization on sections. EdU incorporation was detected at room temperature with the Click-iT^®^ EdU Imaging Kit (Molecular Probes C10339). The staining protocol was optimized for frozen sections using the following modifications: 2 × 10′ PBS-Tween wash, 2 × 10′ 3% BSA incubation, 30′ incubation in the dark with Click-iT^®^ reaction cocktail assembled in the recommended order immediately prior to application, 3% BSA wash, 2x PBS wash.

#### Isolation of embryonic thalamus and single-cell dissociation

The thalamus was manually dissected from control (*Shh*^*ΔSBE1;ΔSBE5/+*^) and mutant (*Shh*^*ΔSBE1;ΔSBE5/ΔSBE1;ΔSBE5*^) brains at four embryonic stages (E12.5, E14.5, E16.5 and E18.5) in ice-cold PBS. The caudal and rostral boundaries of dissection coincided with the cephalic flexure and the mammillary body, respectively. Each thalamus was cut into small pieces and dissociated into a single cell suspension using the Papain Dissociation System (Worthington, LK003153) according to the manufacturer’s protocol. Samples were dissociated in Papain-EBSS solution for 40 min at 37°C. Papain was inactivated with ovomucoid protease inhibitor, and the digested tissue was resuspended in PBS (calcium and magnesium free) containing 0.04% weight/volume BSA (400 μg/mL). Cell suspensions were stained with Trypan Blue to determine the ratio of viable to damaged cells and counted using a haemocytometer (Thermo Fisher Scientific). Single cell suspensions containing more than 90% viable cells were fixed in methanol for 1 week. Samples were rehydrated at a concentration of 700–1200 cells/μL in ice-cold PBS containing 0.04% weight/volume BSA (400 μg/mL) immediately prior to the generation of single-cell RNA sequencing libraries.

#### Single-cell RNA library preparation and sequencing

Single-cell RNA-seq libraries were generated using the 10X Genomics platform (Chromium Single Cell 3′ library and Gel Bead Kit v3, PN-1000075) and sequenced on a NovaSeq 6000 system (Illumina) at the Center for Applied Genomics (Children’s Hospital of Philadelphia). Three independent libraries were generated for each time point and genotype (*n* = 24 libraries).

#### Processing of RNA sequencing data

Fastq files were aligned to the mm10 mouse reference genome and count matrices were generated using the CellRanger (v2.1) pipeline. Except where otherwise specified, we processed and visualized the scRNA-seq counts with the following Seurat-based pipeline, using Seurat v3.0.2 ^70^. We filtered out cells with less than 2,000 UMIs based on the inflection point of the log-transformed barcode rank plot of each sample, or more than 15% of the UMIs coming from mitochondrially encoded genes. In total, 249,071 cells passed these filters, with a median of 4,891 UMIs/cell, (interquartile range: 3,795–6,372 UMIs/cell), 2,466 genes/cell (interquartile range: 2,092–2,943 genes/cell), and 2.7% of UMIs in each cell coming from mitochondrially encoded genes (interquartile range: 2.1–3.5%). We next scaled and centered the UMI counts and used the default vst method to identify the top 2,000 variable genes. We removed all genes from the X and Y chromosomes to reduce the effect of unequal male and female mouse replicates between conditions. To correct for non-biological batch effects between conditions and time points, we used the Harmony algorithm^70^ with its Seurat integration, run on the top principal components (PCs) of the variable genes. Harmony outputs a batch-corrected representation of the scRNA-seq data of same dimensionality as the input PCs. We ran Louvain clustering and UMAP on the output of Harmony to visualize this consolidated gene expression space. The full dataset was visualized using 20 PCs and 3 UMAP dimensions, while the rest of the subset analyses used 20 PCs and 2 UMAP dimensions. To compute differentially expressed genes (DEGs) in the clusters, we used edgeR’s generalized linear model likelihood ratio test (glmLRT)^71^ to compare the gene expression in cells from control mice in a single cluster versus all other clusters in the representation. We also computed DEGs between cells from control and *ΔSBE1/5* mice in each cluster using the same method. To reduce the running time of edgeR, we subsampled large clusters to 2,000 cells when computing DEGs.

#### Annotation of cell populations in the full atlas

We followed the steps outlined above (*Processing of RNA sequencing data*) to visualize and cluster all cells in our scRNA-seq dataset. We used the DEGs in each cluster to annotate it based on cell type, differentiation stage, or area of the brain according to published literature and ISH images from the Allen Developing Mouse Brain Atlas. We did not observe the presence of abundant doublets based on the co-expression of markers.

#### Identification of thalamic nuclei at E18.5

We selected all E18.5 cells from the clusters that we annotated as cTh.N, rTh.N, RT, ZI, and habenula and followed the steps outlined above (*Processing of RNA sequencing data*) to visualize and cluster differentiated cells from thalamic nuclei. We compared DEGs from each cluster to known markers of thalamic nuclei and E18.5 mice ISH data from the Allen Developing Mouse Brain Atlas. We used RayleighSelection^45^ to identify significantly localized genes marking nuclei that could not be disaggregated by unsupervised clustering. Enrichment of each annotated thalamic nucleus in control or *ΔSBE1/5* mice compared to the rest of the E18.5 thalamic clusters was computed using a Fisher’s exact test.

#### Identification of progenitor populations at E12.5

We selected all E12.5 cells from the progenitor cell clusters with *Olig3* expression: cTh.Pro, GABAergic progenitors, and astroglia. We used the same approach described above (processing of RNA sequencing data) to produce higher resolution clusters of just these cells and selected those clusters that had high expression of *Olig3* and *Vim* but did not yet express neuronal differentiation markers (*Neurod1, Stmn2*). We created a UMAP visualization of the resulting 1,885 cells using the top 10 genes that were correlated and the top 10 genes that were anti-correlated with *Nkx2–2, Olig2, Dbx1,* and *Rspo3* (62 genes in total). We associated each progenitor subpopulation (rTh.Pro, cTh.Pro1, cTh.Pro2, epiTh.Pro, cTh.IPC, or preT.Pro) with a subset of these 62 genes based on known markers and a correlation-based hierarchical clustering of the genes. We then assigned each cell to a progenitor subpopulation based on the total counts for each set of genes. Enrichment of each subpopulation in control or *ΔSBE1/5* mice compared to the rest of the E12.5 progenitor clusters was computed using a Fisher’s exact test.

#### Transferring annotations across time points

We transferred thalamic nuclei and progenitor identities across time points by building representations from the clusters cTh.N, rTh.N, RT, ZI, and habenula (for thalamic nuclei), and cTh.Pro, GABAergic progenitors, and astroglia (for progenitors). We processed and clustered these representations following the same approach as in *Processing of RNA sequencing data*. We annotated each cluster, containing cells from all time points, based on the proportion of annotated E18.5 or E12.5 cells, as long as they represented at least 2% of the cells in the cluster. To identify Shh-responsive clusters, we calculated the GSEA score of Shh-responsive genes (*Gli1, Ptch1, Olig2, Nkx2–2, Pdlim3, Fst, Zdbf2, Hs3st1*, and *Slc38a11*) in the list of all genes ordered by fold change in expression between control and *ΔSBE1/5* mice.

#### Thalamic lineages across time

We reconstructed the GABAergic, glutamatergic thalamic, and habenula lineages separately for each time point. For GABAergic lineages, we selected cells from the GABAergic progenitors, rTh.N (early post-mitotic), rTh.N, preTh.N (early post-mitotic), RT, and ZI clusters, as well as the cells labeled as rTh.Pro at E12.5 (*Identification of progenitor populations at E12.5*). For glutamatergic thalamic lineages, we selected cells from the cTh.N and cTh.N (early post-mitotic) clusters, as well as progenitors from all time points in *Olig3*^+^ progenitor clusters. For habenula lineages, we selected cells from the same *Olig3*^+^ progenitor clusters, plus cTh.N (early post-mitotic), habenula, and pretectum clusters. We prepared UMAP representations of these differentiation lineages as described in processing of RNA Sequencing data, with the addition of Seurat’s cell cycle regression to balance out the clear cell cycle effect differences between progenitor and differentiated cells. Cell cycle scores were computed using Seurat’s CellCycleScoring function. We used the velocyto command line interface^49^ and scVelo^50^ to infer and visualize RNA velocity streamlines and pseudotime on the UMAP representations. We identified gene patterns associated with the differentiation trajectory from the top DEG lists, then further identified correlated and anti-correlated genes of interest. The significance of a change in Seurat’s G2/M score between control and *ΔSBE1/5* cells in each cluster was calculated using a Wilcoxon rank-sum test.

#### Locomotor behavior

Locomotor activity was assayed using the force plate actometer as previously described.^73^ Briefly, mice (10–14 weeks of age) were acclimated to the room for 15 min prior to the start of each experiment. Individual mice were placed on an open field plate (28 cm × 28 cm) with four force transducers and sampled at 200 scans/second for 60 min. Each session was digitally recorded. The force plate was wiped down with 70% ethanol after each session. The activity of the mouse was tracked with high accuracy, including total distance traveled, rearing events, low mobility bouts, and time spent in the center 25% of the open field. Data acquisition and calibration procedures were followed as previously described.^74^

#### Rotarod

Balance and coordination were assessed on a five-station Rotarod treadmill (IITC Life Science Inc.). Each mouse was tested three times per day for two consecutive days. All trials lasted for five minutes, the time when maximum speed was reached at a constant rate of acceleration from 4–40 rpm. A trial was terminated when a mouse fell off, made one complete backward revolution while hanging on, or after five minutes. The mice were acclimated to the room for 30 min on each testing day. The machine was wiped down with 70% ethanol in between each trial. Mice (10–14 weeks of age) were tested in four separate cohorts comprising five mice per cohort (n = 10 control and n = 10 *ΔSBE1/5* mutant littermates from 4 separate litters).

#### QUANTIFICATION AND STATISTICAL ANALYSIS

All cell counts in imaging data were performed using the cell counter function in ImageJ (NIH) on tissue sections from at least three control and mutant embryos. In cases where double labeling was examined the tissue was imaged at a single Z-plane. Each channel (green for marker 1, red for marker 2) was first examined independently, assigning a positive count for a given marker to the DAPI stained nucleus most closely associated with the staining. A cell was only counted as double labeled if a single nucleus marked by DAPI had been assigned to the cell labeled by marker1 and marker 2. Statistical analysis of all cell counts was performed in GraphPad Prism using a two-side *t* test. For a given *in situ* probe, expression area was measured from at least three control and mutant embryos using ImageJ software. Quantification of the spatial distribution of genes expressed in the zli was normalized to head size. Statistical analysis of all area and length measurements was performed in GraphPad Prism using a two-sided t test. Differential gene expression analyses of single-cell RNA-seq data were performed using edgeR’s generalized linear model likelihood ratio test (glmLRT).^71^ Control/mutant cell enrichments in single-cell RNA-seq clusters were assessed using a two-sided Fisher’s exact test. Differences in G2/M score between control and *ΔSBE1/5* cells in each single-cell RNA-seq cluster were evaluated using a two-side Wilcoxon rank-sum test. Locomotor, balance, and coordination data was compared between control and *ΔSBE1/5* mice using two-sided t-tests. For t-tests, normality of the data was not tested due to the small sample size. p values and sample sizes for each statistical test are described in the respective figure legend.

## Supplementary Material

1

2

3

4

5

6

## Figures and Tables

**Table:** KEY RESOURCES TABLE

REAGENT or RESOURCE	SOURCE	IDENTIFIER

Antibodies		

Chicken anti-GFP (1:200)	Aves Labs	Cat# GFP-1020; RRID:AB_10000240
Mouse anti-Nkx2.2 (1:300)	DSHB	Cat# 74.5A; RRID:AB_2314951
Rabbit anti-Olig2 (1:300)	Millipore	Cat# AB9610; RRID:AB_570666
Rabbit anti-Foxp2 (1:200)	Abcam	Cat# ab16046; RRID:AB_2107107
Mouse anti-Sox2 (1:100)	R&D Systems	Cat# MAB2018; RRID:AB_358009
Rabbit anti-Sox2 (1:300)	Millipore	Cat# AB5603; RRID:AB_2286686
Rabbit anti-Tyrosine Hydroxylase (1:1000)	Pel-Freez Biologicals	Cat# P40101–0; RRID:AB_461064
Goat anti-Mouse Alexa 488 (1:400)	Thermo Fisher Scientific	Cat# A28175; RRID:AB_2536161
Goat anti-Rabbit Alexa 488 (1:400)	Thermo Fisher Scientific	Cat# A-11008; RRID:AB_143165
Goat anti-Rabbit Alexa 594 (1:400)	Thermo Fisher Scientific	Cat# A-11037; RRID:AB_2534095
Goat anti-Chicken Alexa 488 (1:350)	Thermo Fisher Scientific	Cat# A-11039; RRID:AB_2534096

Critical commercial assays		

Click-iT^®^ EdU Imaging Kit	Molecular Probes	C10339
Papain Dissociation System	Worthington Biochemical Corporation	LK003153
Chromium Single Cell 3′ library and Gel Bead Kit v3 Deposited data	10x Genomics	PN-1000075

Deposited data		

Raw and processed single-cell RNA-seq data	This paper	GEO: GSE211701
Interactive UMAP visualizations	This paper	CELLxGENE: https://cellxgene.cziscience.com/collections/d5cad3f0–56b6–4fbe-8f2b-be92a8c7820f
3D UMAP visualization	This paper	Mendeley Data: https://doi.org/10.17632/kjpj9n66jg (https://data.mendeley.com/datasets/kjpj9n66jg)

Experimental models: Organisms/strains		

*Shh*^*ΔSBE1ΔSBE5/ΔSBE1ΔSBE*5^ (*ΔSBE1/5*)	Yao et al., (2016)^39^	N/A
*Shh-GFP* (429M20eGFP)	Jeong et al., (2006)^67^	N/A
Cbln2-mVenus	Seigneur and Sudhof (2017)^68^	N/A

Software and algorithms		

Cell Ranger (v2.1)	10x Genomics	https://support.10xgenomics.com/single-cell-gene-expression/software/pipelines/latest/what-is-cell-ranger
R (v3.4.1)	The R Foundation	https://cran.r-project.org/
Seurat (v3.0.2)	Stuart et al., 2019^69^	https://satijalab.org/seurat/index.html
Harmony	Korsunsky et al., 2019^70^	https://portals.broadinstitute.org/harmony/
edgeR (v3.20.1)	Robinson et al., 2010^71^	https://bioconductor.org/packages/release/bioc/html/edgeR.html
RayleighSelection	Govek et al., 2019^45^	https://github.com/CamaraLab/RayleighSelection
velocyto command line tool (v0.17.13)	La Manno et al., 2018^49^	http://velocyto.org/
Python (v3.9.10)	Python Software Foundation	https://www.python.org/
scvelo (v0.2.4)	Bergen et al., 2020^50^	https://scvelo.readthedocs.io/

Other		

Leica TCS SP8 MP system	Leica	https://www.leica-microsystems.com/products/confocal-microscopes/p/leica-tcs-sp8-mp/downloads/
Leica DM 5500 upright microscope system	Leica	https://www.leica-microsystems.com/products/light-microscopes/p/leica-dm5500-b/

## Highlights

- Molecular signatures distinguish most thalamic nuclei prior to birth
- Thalamic nuclei emerge from four distinct cell lineages
- Defects in motor thalamic nuclei formation cause infantile Parkinson’s disease
