# Supplementary material for: Developmental trajectories of thalamic progenitors revealed by single-cell transcriptome profiling and Shh perturbation
Source: Cell Rep. Author manuscript; Available in PMC 2023 Jan 27. (PMC9880597; doi:10.1016/j.celrep.2022.111768)
Supplement: 1 [file NIHMS1855850-supplement-1.pdf]

Cell Reports, Volume *41*

Supplemental information

Developmental trajectories of thalamic progenitors revealed by single-cell transcriptome profiling and Shh perturbation

Kiya W. Govek, Sixing Chen, Paraskevi Sgourdou, Yao Yao, Steven Woodhouse, Tingfang Chen, Marc V. Fuccillo, Douglas J. Epstein, and Pablo G. Camara

Supplemental Figures

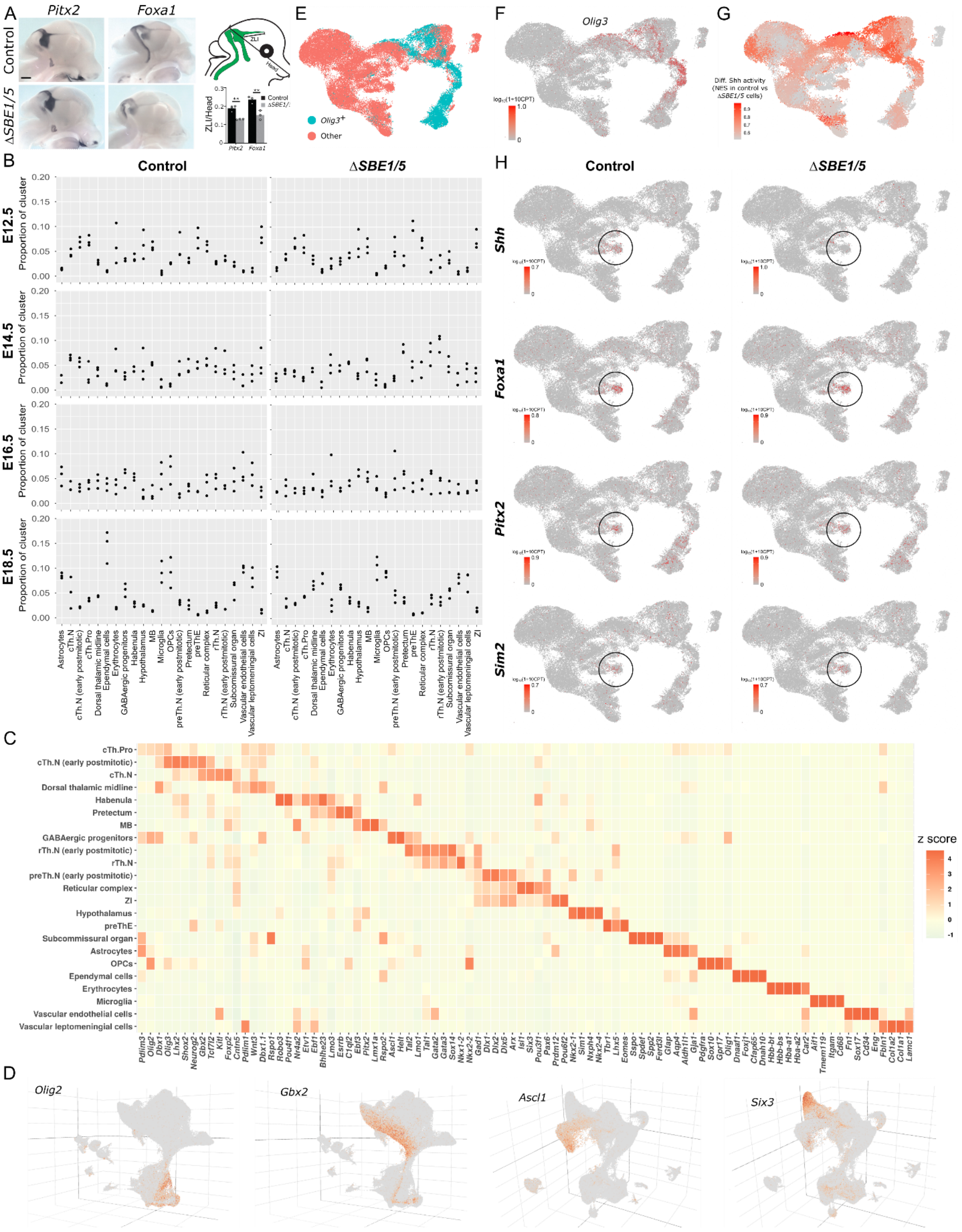

Figure S1. Annotation and evaluation of the single-cell transcriptomic atlas of the developing caudal diencephalon, Related to Figure 1. (A) The expression of ZLI markers is maintained in *ΔSBE1/5* mutants. Whole-mount RNA in situ hybridization for *Pitx2 and Foxa1* on bisected heads from control (top) and *ΔSBE1/5* embryos (bottom) at E12.5 showing expression in the ZLI. The area of gene expression in the ZLI, normalized to head size, is depicted at right in control and mutant embryos (**: $p < 0.01$, two-sided t-test, $n = 3$ mice of each condition, error bars represent standard deviation). Scale bar = 500μm. **(B)** Cells from independent biological replicates are similarly delineated across all cell populations. The plots represent the proportion of cells from each of the 24 mice in each cluster. **(C)** Annotation of the single-cell transcriptomic atlas. Heat-map depicting the expression of some of the differentially expressed genes that were used to annotate the cell populations of Figure 1D. **(D)** The single-cell RNA-seq atlas contains continuous developmental trajectories associated with glutamatergic and GABAergic thalamic neurons. The 3D UMAP representation of the single-cell RNA-seq dataset is colored by the expression level of selected cTh.Pro (*Olig2*) and rTh.Pro (*Ascl1*) thalamic progenitors and cTh.N (*Gbx2*) and rTh.N (*Six3*) postmitotic thalamic neurons. **(E)** *Olig3*$^{+}$ progenitor cell subpopulation shows a downregulation of Shh responsive genes in *ΔSBE1/5* compared to control cells. UMAP representation of progenitor cells from all timepoints and conditions indicating the population of *Olig3*$^{+}$ progenitors. **(F, G)** The UMAP is colored by the expression of *Olig3* (F) and the normalized enrichment score (NES) of Shh-responsive genes (*Gli1*, *Ptch1*, *Olig2*, *Nkx2-2*, *Pdlim3*, *Fst*, *Zdbf2*, *Hs3st1*, and *Slc38a11*) in control vs *ΔSBE1/5* cells (G), demonstrating the downregulation of Shh-responsive genes in *Olig3*$^{+}$ progenitors from

ΔSBE1/5 embryos. **(H)** The single-cell RNA-seq data is consistent with a depletion of *Shh* expression in the ZLI of *ΔSBE1/5* mutants. The region corresponding to the ZLI is indicated in the UMAP representation of progenitor cells from all timepoints and conditions. The UMAP is colored by the gene expression levels of *Shh* and selected transcription factors that are co-expressed in the ZLI (*Foxa1, Pitx2, Sim2*). The expression of *Shh* is downregulated in the ZLI cells of *ΔSBE1/5* embryos. However, other ZLI markers maintain their expression in *ΔSBE1/5* embryos. CPT: counts per thousand.

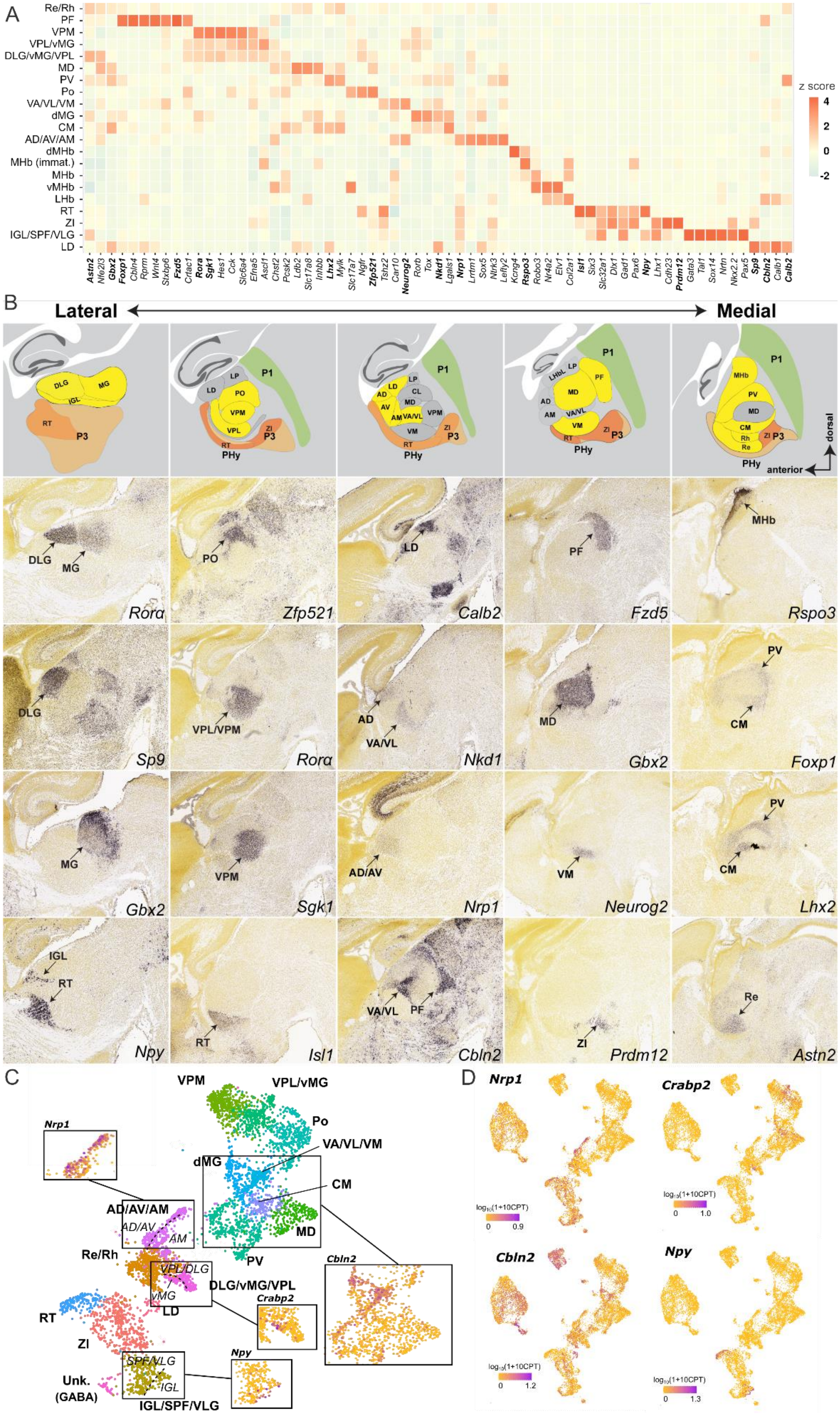

A
Re/Rh
PF
VPM
VPL/vMG
DLG/vMG/VPL
MD
PV
Po
VA/VL/VM
dMG
CM
AD/AV/AM
dMHb
MHb (immat.)
MHb
vMHb
LHb
RT
ZI
IGL/SPF/VLG
LD
z score
4
2
0
-2
Astn2 Nfe2l3 Gbx2 Foxp1 Cbln4 Rprm Wnt4 Stxbp6 Fzd5 Crtac1 Rora Sgk1 Hes1 Cck Slc6a4 Efna5 Ascl1 Chst2 Pcsk2 Ldb2 Slc17a8 Inhbb Lhx2 Mylk Slc17a7 Ngfr Zfp521 Tshz2 Car10 Neurog2 Rorb Tox Nkd1 Lgals1 Nrp1 Lrrtm1 Sox5 Ntrk3 Lefty2 Kcng4 Rspo3 Robo3 Nr4a2 Elf1 Col2a1 Isl1 Six3 Slc32a1 Dlx1 Gad1 Pax6 Npy Lhx1 Cdh23 Prdm12 Gata3 Tal1 Sox14 Nrtn Nkx2.2 Pax5 Sp9 Cbln2 Calb1 Calb2
B
Lateral
Medial
P1
P3
PHy
dorsal
anterior
DLG MG Rora
PO Zfp521
LD Calb2
PF Fzd5
MHb Rspo3
DLG Sp9
VPL/VPM Rora
AD VA/VL Nkd1
MD Gbx2
PV CM Foxp1
MG Gbx2
VPM Sgk1
AD/AV Nrp1
VM Neurog2
PV CM Lhx2
IGL RT Npy
RT Isl1
VA/VL PF Cbln2
ZI Prdm12
Re Astn2
C
VPM
VPL/vMG
Po
VA/VL/VM
dMG
CM
MD
PV
AD/AV/AM
AD/AV
AM
Re/Rh
VPL/DLG
vMG
DLG/vMG/VPL
LD
RT
ZI
Unk.
(GABA)
SPF/VLG
IGL
IGL/SPF/VLG
Nrp1
Cbln2
Crabp2
Npy
D
Nrp1
Crabp2
Cbln2
Npy
log10(1+10CPT)

Figure S2. Annotation of E18.5 neuronal thalamic populations in the single-cell RNA-seq data, Related to Figure 2. (A) Heat-map depicting the expression of marker genes from the Allen Brain Developmental Mouse Atlas that were used to annotate the cell subpopulations identified in the E18.5 control cells from the postmitotic cTh.N, rThN, ZI, habenula, and reticular complex clusters. Representative genes for which RNA in situ hybridization data is show in (B) are indicated in bold. **(B)** (Top row) Schematic representation of sagittal sections through the diencephalon at E18.5 displaying pretectum (green), thalamus (yellow) and prethalamus (brown/beige). (Bottom rows) RNA in situ hybridization data from the Allen Developing Mouse Brain Atlas at E18.5 for select differentially expressed genes from each of the single-cell subpopulations. Image credit: Allen Institute. Arrows point to thalamic nuclei showing expression of genes indicated in each panel. **(C)** Transcriptomic signatures of closely related thalamic nuclei. We used a spectral graph method to dissect the transcriptional heterogeneity within the cell populations identified in the clustering analysis of E18.5 thalamic nuclei. Several regions of the single-cell RNA-seq UMAP representation of E18.5 thalamic nuclei are highlighted and colored by the expression level of marker genes identified by this method. **(D)** The complete single-cell RNA-seq UMAP representation of E18.5 thalamic nuclei is colored by the expression level of marker genes identified by this method. CPT: counts per thousand.

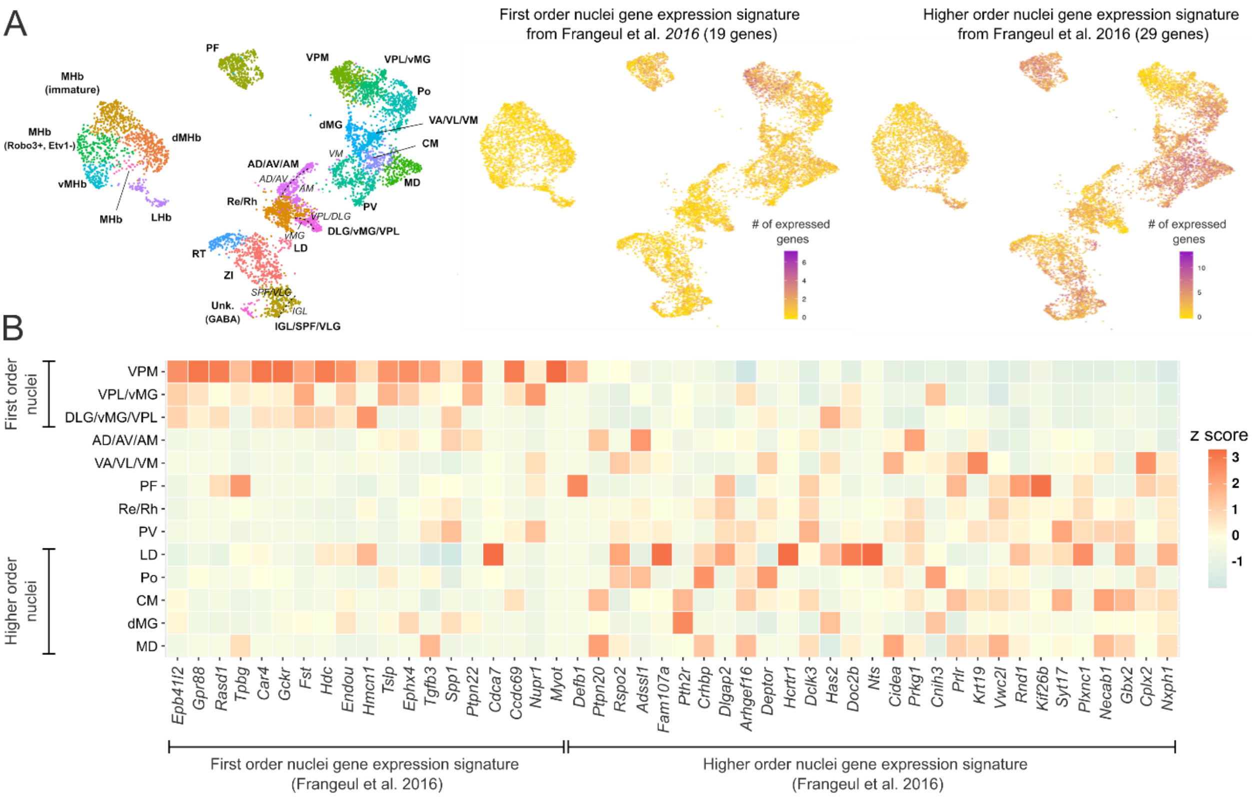

Figure S3. Postnatal gene expression signatures of first- and higher-order nuclei are evident at E18.5, Related to Figure 2. (A) The single-cell RNA-seq UMAP representation of E18.5 thalamic nuclei is colored by the number of expressed genes belonging to the postnatal gene expression signatures of first order and higher order nuclei from Frangeul *et al.* (2016). For reference, the location of the different thalamic nuclei cell populations identified in this study is also indicated in the UMAP. **(B)** Heatmap showing the expression of postnatal first order and higher order thalamic nuclei gene expression signatures of Frangeul et al. [S1] in E18.5 thalamic nuclei.

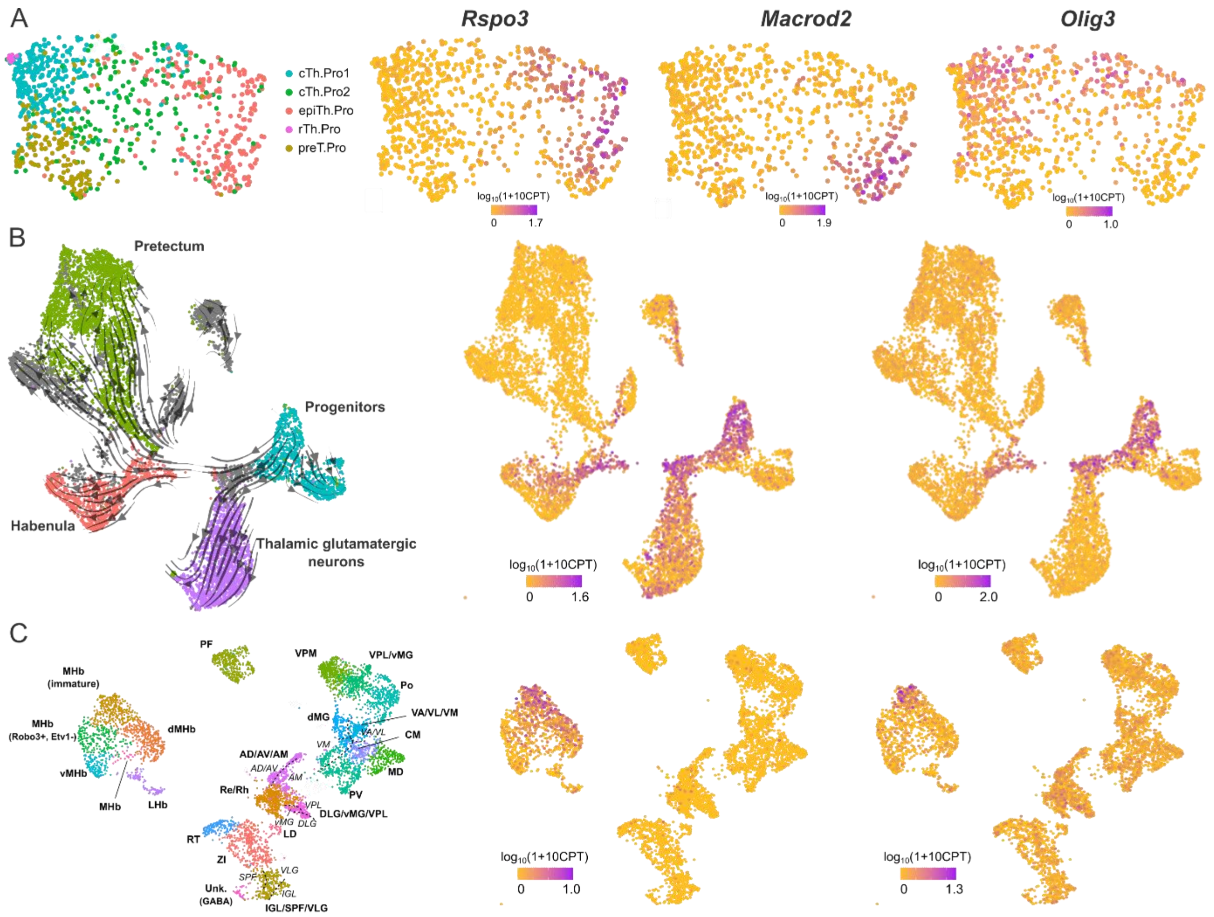

Figure S4. The expression of several epiTh.Pro progenitor marker genes is maintained across the acquisition of habenular nuclei neuronal identities, Related to Figure 2. (A) UMAP representation of the mRNA expression data of E12.5 control cells from the *Olig3*$^{+}$ progenitor population colored by the expression levels of *Rspo3, Macrod2,* and *Olig3*. The expression of *Rspo3* and *Macrod2* is upregulated in epiTh.Pro progenitors with low *Olig3* expression. **(B)** UMAP representation of E14.5 control cells, comprising *Olig3*$^{+}$ progenitors and early postmitotic neurons from the glutamatergic thalamus, pretectum, and habenula, colored by the expression of *Rspo3* and *Macrod2.* The expression of these genes is maintained in early post-mitotic cells of the habenula. The RNA velocity field is also shown for reference. **(C)** UMAP representation of E18.5

thalamic nuclei colored by the expression of *Rspo3* and *Macrod2,* showing the expression of these genes in immature neurons of the habenula. CPT: counts per thousand.

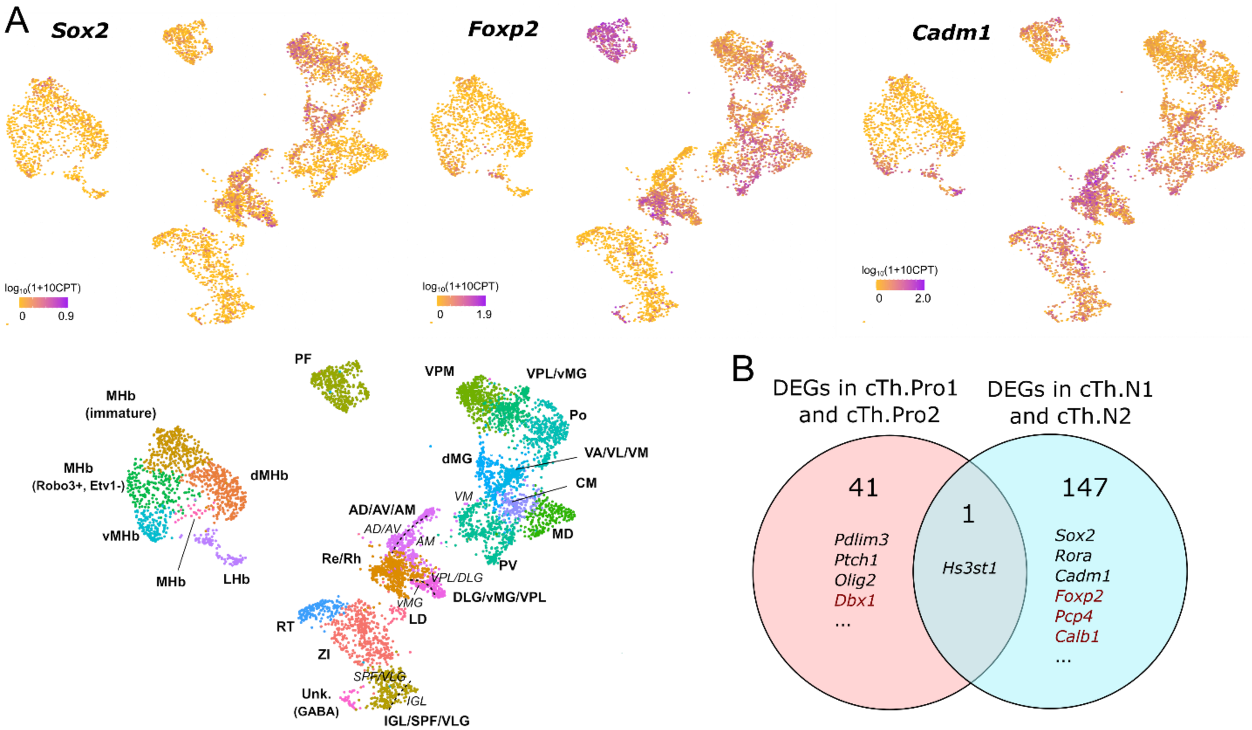

Figure S5. Differentially expressed genes between glutamatergic thalamic lineages, Related to Figure 3. (A) Expression of *Sox2*, *Foxp2*, and *Cadm1* lineage markers in E18.5 thalamic nuclei. The single-cell RNA-seq UMAP representation of E18.5 thalamic nuclei cells from control mice is colored by the expression level of *Sox2*, *Foxp2*, and *Cadm1*. For reference, the location of the different thalamic nuclei cell populations identified in this study is also indicated in the UMAP (bottom). CPT: counts per thousand. **(B)** Most of the genes expressed in glutamatergic thalamic progenitor cells ceased to be expressed after mitotic arrest. Venn diagram showing the number of genes that are differentially expressed (fold-change > 2, generalized linear model likelihood ratio test adjusted *p*-value < 0.05) between cTh.Pro1 and cTh.Pro2 progenitors (pink circle), and between cTh.N1 and cTh.N2 post-mitotic neurons (blue circle). Only *Hs3st1*, which marks cTh.Pro1 progenitors, continues to be differentially expressed in cTh.N1 neurons. Genes that are upregulated in cTh.Pro1 and/or cTh.N1

are marked in black, whereas genes that are upregulated in cTh.Pro2 or cTh.N2 are marked in red. See also Tables S4 and S5.

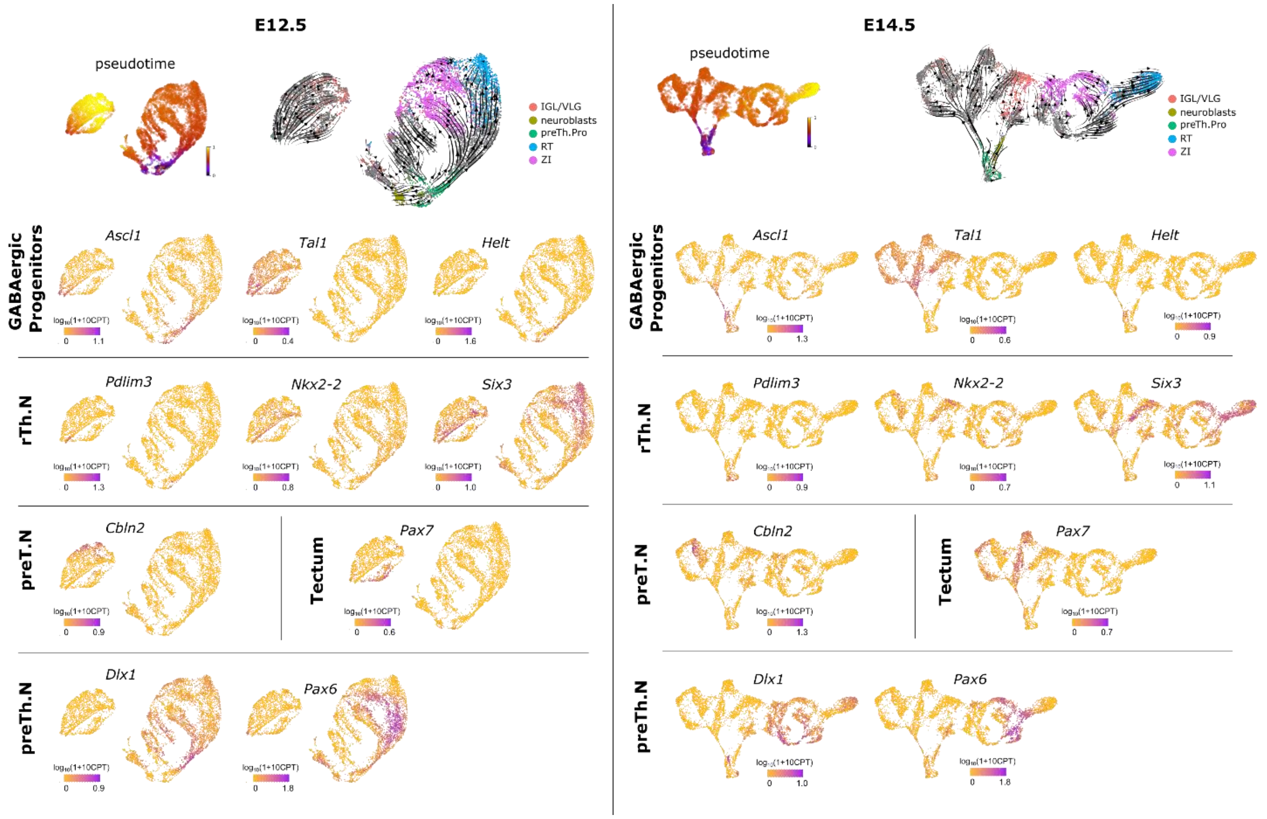

(cont. on next page)

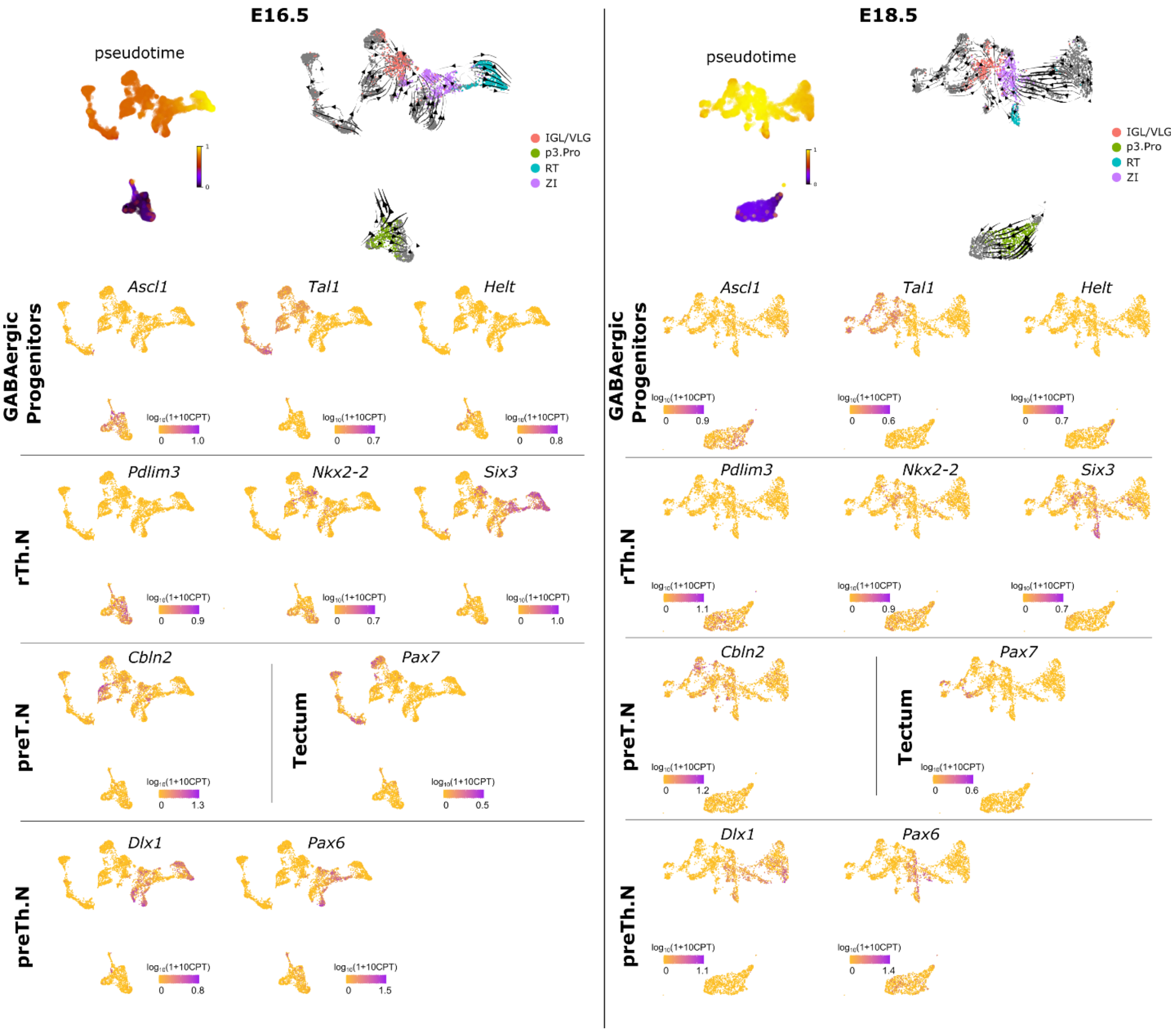

Figure S6. GABAergic neurons diverge from progenitors with a shared transcriptional identity, Related to Figure 4. UMAP representation and RNA velocity field of the single-cell RNA-seq data of the GABAergic cell lineages in control embryos at each developmental stage. For reference, the same UMAP is also colored by the inferred cell differentiation pseudo-time and the gene expression levels of several marker genes associated with distinct lineages. *Tal1*$^{+}$ progenitors lead to three distinct lineages that are marked by the expression of *Six3*, *Cbln2*, and *Pax7* and give rise to GABAergic neurons of the IGL/VLG thalamic nuclei, the pretectum, and the tectum,

respectively. In contrast, GABAergic neuronal populations of the ZI and RT originate from a different lineage characterized by the early expression of *Dlx1* followed by *Pax6* expression. CPT: counts per thousand.

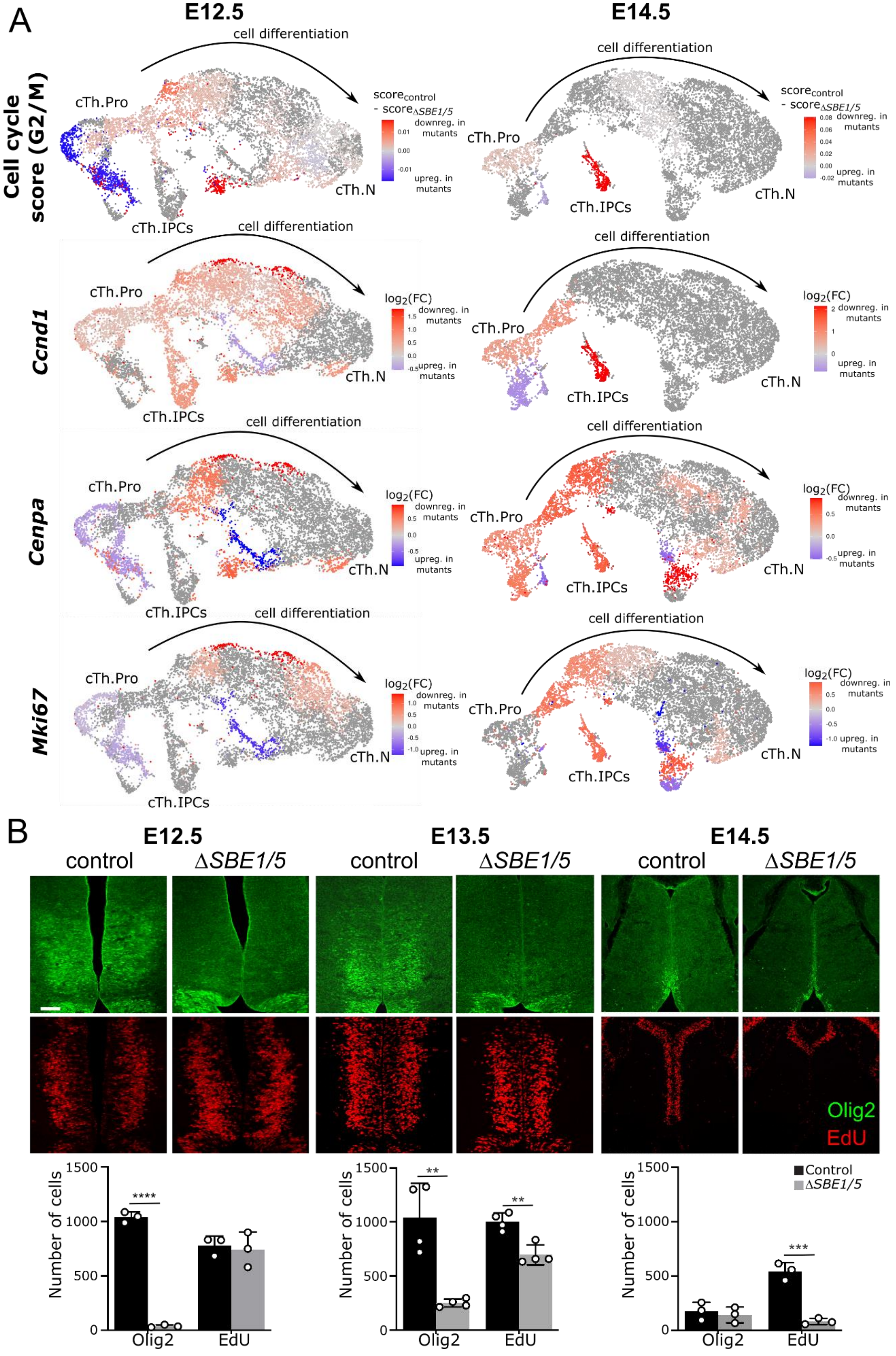
A
E12.5
E14.5
cell differentiation
Cell cycle score (G2/M)
cTh.Pro
cTh.IPCs
cTh.N
score control - score ΔSBE1/5
downreg. in mutants
upreg. in mutants
Ccnd1
log2(FC)
Cenpa
Mki67
B
E12.5
E13.5
E14.5
control
ΔSBE1/5
Olig2
EdU
Number of cells
Control
ΔSBE1/5
Olig2
EdU

Figure S7. Cell cycle is downregulated in *ΔSBE1/5* glutamatergic progenitors, Related to Figure 4. (A) Differential gene expression analysis between control and *ΔSBE1/5* glutamatergic progenitor cells reveals the downregulation of cell cycle genes in mutant cells. The single-cell RNA-seq UMAP representation of the glutamatergic thalamic cell lineage at E12.5 (left) and E14.5 (right) is colored by the difference in the G2/M cell cycle gene expression score between control and *ΔSBE1/5* cells (top), and the fold-change (FC) in the expression of individual cell cycle genes. The analysis shows G2/M cell cycle genes are downregulated in thalamic progenitors and IPCs in *ΔSBE1/5* embryos. **(B)** EdU incorporation and immunofluorescence staining for Olig2 in coronal sections of the thalamus in control and *ΔSBE1/5* embryos. The figure shows a depletion of Olig2 expressing cells (cTh.Pro1 cells) and a reduction in the number of EdU incorporating cells over time in *ΔSBE1/5* embryos (**: $p < 0.01$, ***: $p < 0.001$, ****: $p < 0.0001$; two-sided t-test, $n = 3$ mice of each condition for E12.5 and E14.5, $n = 4$ mice of each condition for E13.5, error bars represent standard deviation).

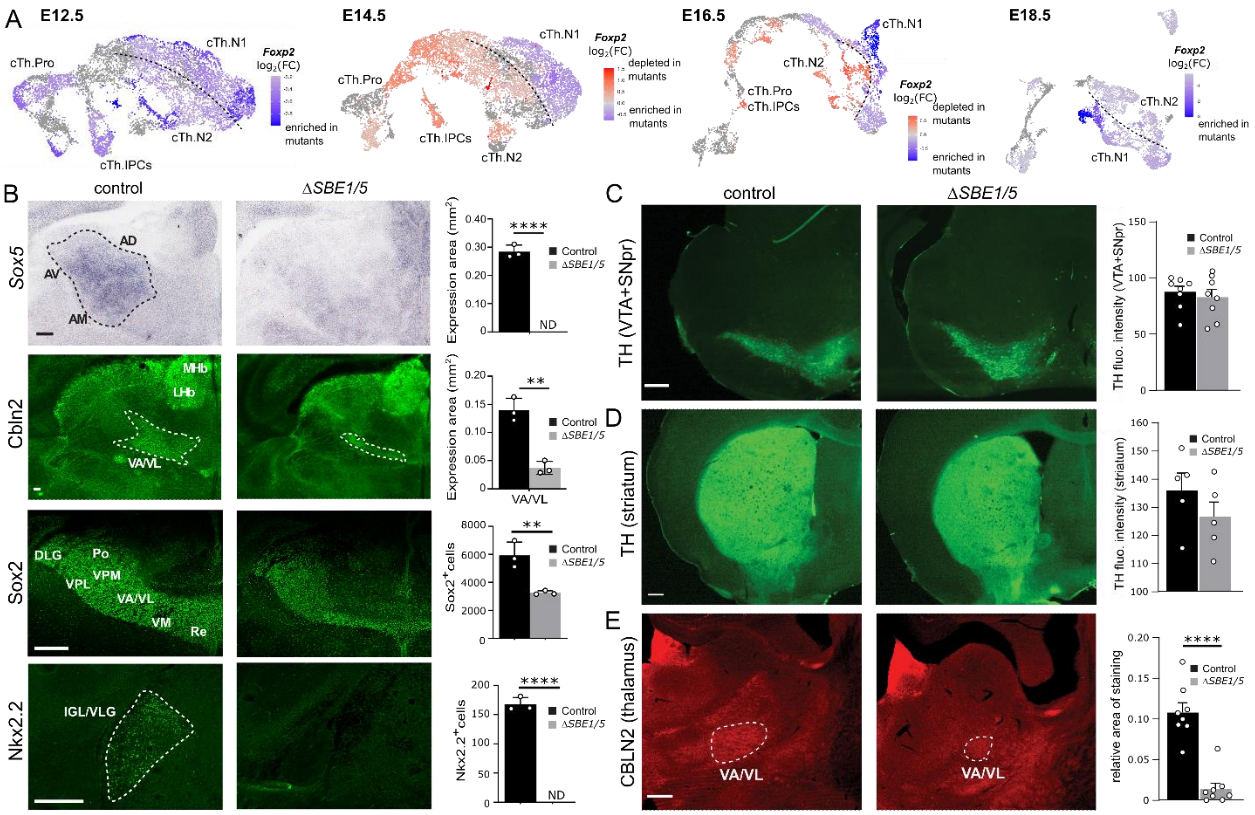

Figure S8. Motor thalamic nuclei are compromised in neonatal and adult *ΔSBE1/5* mice, Related to Figure 5. (A) Postmitotic *Foxp2*$^{+}$ thalamic neurons are enriched in *ΔSBE1/5* mice. Fold-change in the proportion of *Foxp2*$^{+}$ cells during glutamatergic thalamic nuclei differentiation in control and *ΔSBE1/5* mice for each timepoint, showing an enrichment for Foxp2+ cells in the cTh.N1 lineage. **(B)** Shh-dependent nuclei are substantially reduced in newborn *ΔSBE1/5* mice. RNA in situ hybridization (Sox5) and immunostaining for Cbln2 (Cbln2-Venus), Sox2 and Nkx2.2 on coronal sections of the thalamus from control and *ΔSBE1/5* mice at P0, showing a large reduction in the area of staining (Sox5, Cbln2) and number of cells (Sox2, Nkx2.2) within Shh-dependent thalamic nuclei, consistent with the results of the single-cell RNA-seq analysis (**: $p < 0.01$, ****: $p < 0.0001$, two-sided t-test, $n = 3$ mice of each condition, error bars represent standard deviation). Scale bars = 100μm. **(C)** Motor control regions are

unaffected in the midbrain but reduced in the thalamus of adult *ΔSBE1/5* mice. TH immunostaining in the substantia nigra and ventral tegmental area shows no significant difference between *ΔSBE1/5* and control mice (p = 0.485, n = 3, 2-4 sections/brain, two-sided t-test). **(D)** TH immunostaining in the striatum is unaffected in *ΔSBE1/5* compared to control mice (p = 0.075, n = 3, 1-2 sections/brain, two-sided t-test). **(E)** Cbln2 immunostaining in VA/VL motor thalamic nuclei is significantly reduced in *ΔSBE1/5* compared to control mice ($p < 0.0001$, n = 3, 2-3 sections/brain, two-sided t-test). Scale bars= 300 μm. Age of mice: 3-5 months.

Supplemental Methods

Methods S1. Analysis of shared genetic programs across distinct sensory modalities during embryogenesis

Sensory inputs in the brain follow cortico-thalamo-cortical loops, where first order thalamic nuclei project peripheral sensory inputs onto the primary sensory cortex, and higher order thalamic nuclei receive their input from the primary sensory cortex and project it back to a secondary cortex [S2-S4]. The hierarchical position that each nucleus occupies in these circuits has been shown to be the primary determinant of the postnatal transcriptional identity of somatosensory, visual and auditory thalamic nuclei [S1]. To assess whether the shared transcriptional programs between same-order nuclei are established earlier during embryogenesis, we projected the postnatal day 3 (P3) differential gene expression signatures of first- and higher-order nuclei from a previous study [S1] onto our single-cell gene expression data of E18.5 thalamic nuclei. This analysis revealed that postnatal gene expression signatures of first- and higher-order nuclei are already present at E18.5 (Figures S3A and S3B). We observed high expression of genes that are postnatally associated with first-order nuclei in the VPL, VPM, and vMG cell populations, and of genes that are postnatally associated with higher-order nuclei in the Po, parafascicular (PF), centromedian (CM), medial dorsal (MD), lateral dorsal (LD), and dMG transcriptomic cell subpopulations (Figures S3A and S3B), in agreement with the hierarchical order of sensory thalamic nuclei in cortico-thalamo-cortical loops [S4]. These results indicate that the transcriptional signatures of first- and higher-order nuclei are initiated during embryogenesis and suggests that the input-dependent logic of first order and higher order thalamic nuclei acts on a pre-

specified transcriptional program that initiates at embryonic stages of thalamic development and continues to be refined into the early postnatal period. As neuronal connections between thalamic nuclei and their cortical and subcortical targets continue to mature, additional gene expression networks are likely to emerge that further distinguish differences or consolidate similarities between thalamic nuclei [S5, S6].

Methods S2. Analysis of GABAergic transcriptional lineages

To identify the molecular logic that drives the generation of a diverse pool of inhibitory neurons in the thalamus, we performed an analysis of the GABAergic cell populations similar to the one described above for glutamatergic cell lineages. We constructed separate single-cell gene expression representations for GABAergic cells (GABAergic progenitors, rTh.N/rostral thalamic neurons, preTh.N/prethalamic neurons early post-mitotic, ZI, and RT clusters) and computed the RNA velocity vector field in these representations for each time point (Figure S6). GABAergic progenitors expressed the bHLH transcription factor *Ascl1* and comprised two distinct transcriptomic cell lineages (Figure S6). One of the cell lineages was characterized by the early expression of *Dlx1* followed by *Pax6* expression. Upon further differentiation, this lineage led to the GABAergic neuronal populations of the ZI and RT (Figure S6). The other cell lineage originated from *Tal1*$^+$ progenitors and split into three distinct sub-lineages that were marked by the expression of *Six3*, *Cbln2*, and *Pax7* (Figure S6). These sub-lineages led respectively to the GABAergic neurons of the IGL/VLG thalamic nuclei, the pretectum, and the tectum. The thalamic sub-lineage was also characterized by the co-expression of Shh-responsive genes, such as *Pdlim3* and *Nkx2-2*, suggesting the involvement of regional Shh signaling in its specification (Figure S6). Although these results are based

on the continuity of gene expression programs across cell differentiation and cannot be relied upon to define clonal relationships, they are consistent with recent clonal studies of the mouse forebrain and midbrain showing a common progenitor origin for GABAergic neurons with drastically different transcriptomic profiles and anatomical locations [S7, S8]. We conclude that GABAergic neurons of the thalamus derive from *Tal1*$^+$ neural progenitors and are characterized by the expression of Shh-responsive genes.

References

S1. Frangeul, L., Pouchelon, G., Telley, L., Lefort, S., Luscher, C., and Jabaudon, D. (2016). A cross-modal genetic framework for the development and plasticity of sensory pathways. Nature *538*, 96-98. 10.1038/nature19770.

S2. Herkenham, M. (1980). Laminar organization of thalamic projections to the rat neocortex. Science *207*, 532-535. 10.1126/science.7352263.

S3. Sherman, S.M., and Guillery, R.W. (1998). On the actions that one nerve cell can have on another: distinguishing "drivers" from "modulators". Proceedings of the National Academy of Sciences of the United States of America *95*, 7121-7126. 10.1073/pnas.95.12.7121.

S4. Sherman, S.M., and Guillery, R.W. (2006). Exploring the thalamus and its role in cortical function (MIT press).

S5. Phillips, J.W., Schulmann, A., Hara, E., Winnubst, J., Liu, C., Valakh, V., Wang, L., Shields, B.C., Korff, W., Chandrashekar, J., et al. (2019). A repeated molecular architecture across thalamic pathways. Nat Neurosci *22*, 1925-1935. 10.1038/s41593-019-0483-3.

S6. Roy, D.S., Zhang, Y., Halassa, M.M., and Feng, G. (2022). Thalamic subnetworks as units of function. Nat Neurosci. 10.1038/s41593-021-00996-1.

S7. Bandler, R.C., Vitali, I., Delgado, R.N., Ho, M.C., Dvoretskova, E., Ibarra Molinas, J.S., Frazel, P.W., Mohammadkhani, M., Machold, R., Maedler, S., et al. (2021). Single-cell delineation of lineage and genetic identity in the mouse brain. Nature. 10.1038/s41586-021-04237-0.

S8. Jager, P., Moore, G., Calpin, P., Durmishi, X., Salgarella, I., Menage, L., Kita, Y., Wang, Y., Kim, D.W., Blackshaw, S., et al. (2021). Dual midbrain and forebrain origins of thalamic inhibitory interneurons. Elife *10*. 10.7554/eLife.59272.
